# Setd2 ensures the establishment of a precise basal inflammatory state within murine hematopoietic stem/progenitor cells

**DOI:** 10.1038/s41419-025-08110-0

**Published:** 2025-11-06

**Authors:** Hong Tao, Xinyue Luo, Jiachun Song, Fuhui Wang, Yinyin Xie, Rong Fan, Xiaojian Sun, Qiuhua Huang, Yuanliang Zhang

**Affiliations:** Shanghai Institute of Hematology, State Key Laboratory of Medical Genomics, National Research Center for Translational Medicine at Shanghai, Ruijin Hospital Affiliated to Shanghai Jiao Tong University School of Medicine, Shanghai, China

**Keywords:** Inflammation, Haematopoietic stem cells

## Abstract

The maintenance of a basal immunoinflammatory signature in hematopoietic stem/progenitor cells (HSPCs) constitutes a fundamental regulatory axis governing hematopoietic competence and immune effector generation. While epigenetic repressors constrain this inflammatory phenotype, the molecular amplifiers that preserve this critical state remain undefined. Through integrated single-cell transcriptomic/epigenomic profiling and functional interrogation, we identify Setd2-mediated H3K36me3 as an indispensable epigenetic amplifier sustaining baseline inflammation in murine HSPCs. *Setd2* ablation specifically eliminated interferon (IFN)-enriched HSPC subpopulations and attenuated inflammatory signaling cascades. Functionally, *Setd2*-deficient HSPCs exhibited impaired IFNγ responsiveness, compromised B-lymphopoiesis, and diminished reconstitution capacity due to Lin^−^c-Kit^+^Sca1^high^ cell depletion. Paradoxically, *Setd2* loss conferred resistance to IFNγ-induced HSPCs exhaustion, which may contribute to the maintenance of *Setd2*-deficient HSPCs in our myelodysplastic syndrome (MDS) model under the inflammatory milieu. Mechanistically, Setd2 sustained chromatin accessibility and enhancer (H3K27ac) activity at inflammatory gene loci. This work delineates a critical link between Setd2-mediated chromatin regulation, baseline inflammation, HSPC function, and immune competence, providing insights into inflammatory dysregulation in hematopoietic malignancies like MDS.

## Introduction

Hematopoietic stem cells (HSCs) function as frontline sentinels through their constitutive expression of pattern recognition and cytokine receptors, poising them as primary responders to infection [1]. This sensing capacity drives HSC activation from quiescence to generate emergency myelopoiesis, while commensal microbiota-derived inflammatory signals calibrate basal self-renewal and differentiation programs under homeostasis [1]. Single-cell sequencing analysis reveals tonic interferon (IFN) signaling persists in HSCs even in sterile conditions [2], with developmental studies demonstrating essential roles for pro-inflammatory signals (e.g., TNFα-NF-κB pathway, IFNγ) in HSC ontogeny [3–5]. However, dysregulated inflammatory signaling precipitates hematopoietic pathologies ranging from bone marrow (BM) failure to leukemic transformation [6–9], highlighting the critical balance of basal inflammatory tone in HSCs.

Genetic lesions in pattern recognition receptors (PRRs), cytokine signaling components, and downstream mediators induce maladaptive immunoinflammatory activation in hematopoietic stem/progenitor cells (HSPCs) [10], inducing transformation to myelodysplastic syndromes (MDSs) [7]. Parallel disruptions in alternative splicing [11], ubiquitin-proteasome system [12], and other processes also potentiate inflammatory dysregulation in HSPCs—epigenetic factors like TET2 and ASXL1, frequent mutation targets in myeloid neoplasms, demonstrate different roles in HSPC inflammation regulation [13, 14]. *Tet2* deficiency hyperactivates IL-6/Shp2/Stat3/Morrbid and STING signaling pathways, conferring apoptosis resistance and enhancing self-renewal of HSPCs [15, 16]. *Asxl1* mutation generates paradoxical inflammatory signatures by co-upregulation of pro- and anti-inflammatory genes, thereby promoting the clonal fitness of HSPCs [17]. Emerging evidence suggests that *ASXL1* mutation may exacerbate inflammation in myeloproliferative neoplasms via the EGR1-TNFA axis in HSPCs [18]. Despite multiple epigenetic modifiers constraining inflammatory signaling in HSPCs, it remains unclear whether a dedicated epigenetic amplifier exists to maintain a basal inflammatory state in hematopoietic compartments.

SETD2 catalyzes histone H3K36 trimethylation within gene bodies [19–21], with frequent mutations observed across malignancies [22]. Murine studies (including our work) establish *Setd2* as a critical tumor suppressor in hematopoiesis [23, 24]. In addition, *SETD2* mutations tend to remodel tumor microenvironment and facilitate immune evasion [25]. Beyond oncogenesis, scattered reports associate *SETD2* mutation with primary immunodeficiency disorders (PIDs) [26], paralleling murine findings demonstrating its necessity in lymphocyte development (e.g., B cells, GATA3^+^ST2^+^ regulatory T cells, and type 3 innate lymphoid cells (ILC3)) [27–29] and dextran sodium sulfate (DSS)-induced intestinal inflammation [30]. Mechanistic studies reveal SETD2-mediated STAT1 methylation as a key modulator of hepatic antiviral responses [31], consolidating its status as a multifunctional immune regulator. Nevertheless, the potential role of SETD2 in modulating inflammatory signaling within HSPCs remains to be elucidated.

Our investigation revealed that *Setd2* deletion attenuated baseline inflammation in HSPCs, especially IFN activity, critically impairing the maintenance of Lin^−^c-Kit^+^Sca1^high^ cells (Sca1^high^-LSKs). Consequently, we investigated the epigenetic mechanisms underlying impaired baseline inflammatory signaling in HSPCs, along with its potential impact on HSPC functional regulation.

## Results

### *Setd2* is indispensable for maintaining the basal inflammatory state in murine HSPCs

**Fig. 1:**
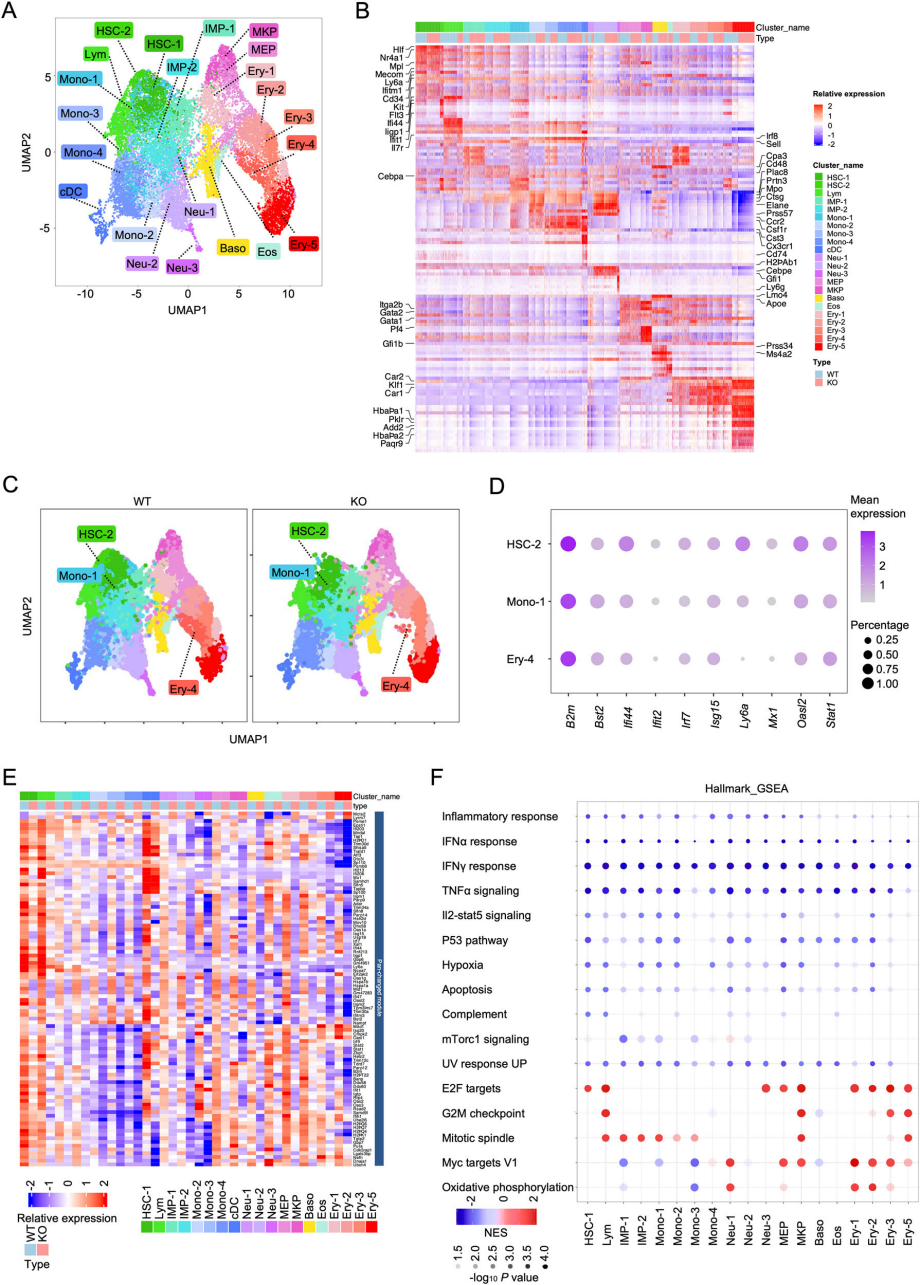
*Setd2* deficiency suppresses intrinsic IFN and inflammatory response gene expression in HSPCs. **A** UMAP projection of BM LKs from WT and KO mice. Annotated populations: HSC, Lym, IMP, Mono, classical dendritic cell progenitor (cDC), Neu, megakaryocyte-erythroid progenitor (MEP), MKP, basophil progenitor (Baso), eosinophil progenitor (Eos), and Ery. **B** Heatmap of Z-score normalized expression for cluster-defining marker genes across 22 populations. Genes and clusters are ordered by hierarchical clustering. **C** UMAP visualization of WT (left) and KO (right) LKs. Cluster HSC-2 (green), Mono-1 (blue), and Ery-4 (red) were nearly absent in the KO group. **D** Bubble plot showing expression of IFN signaling genes in cluster HSC-2, Mono-1, and Ery-4. Bubble size indicates the percentage of cells expressing the genes; color intensity indicates the mean normalized expression level. **E** Heatmap of pseudo-bulk gene expression profiles for the Pan-changed module (hierarchically clustered DEGs; see Table S3). Color scale: relative expression (blue: low; red: high). **F** GSEA of hallmark gene sets enriched in each cluster of KO versus WT LKs. Bubble color: Normalized Enrichment Score (NES; red: KO-enriched, blue: WT-enriched); Brightness: −log₁₀ *P* value; Size: number of core enriched genes (FDR < 0.05).

In our previous work, we demonstrated that *Setd2* deficiency impairs HSC self-renewal, induces skewed hematopoietic differentiation, and results in the manifestation of MDS symptoms [23]. However, the intrinsic mechanisms of hematopoietic dysfunction remain to be elucidated. Therefore, we performed single-cell RNA sequencing (scRNA-seq) on BM Lin^−^c-Kit^+^ cells (LKs) from polyinosinic-polycytidylic acid (poly (I: C))-induced wild-type (WT) and *Setd2*^fl/fl^ knockout (KO) mice (3 weeks post-induction, Fig. S1A, B). In all, 8 457 WT cells and 9 510 KO cells with comparable numbers of genes and unique molecular identifiers (UMIs) underwent Uniform Manifold Approximation and Projection (UMAP)-based clustering (Fig. S1C). A total of 22 lineage-defined clusters were identified, including HSC, immature myeloid progenitor (IMP) [32], lymphoid progenitor (Lym), monocyte progenitor (Mono), neutrophil progenitor (Neu), and erythroid progenitor (Ery) [33] (Figs. 1A, B and S1D, Table S1). Following *Setd2* deletion, cluster distribution was shifted with marked reductions in Lym (WT vs. KO: 8.44% vs. 3.13%, *P* = 6.22e-48) and Mono (24.72% vs. 18.00%, *P* = 7.26e-19), and expansions in IMP (9.61% vs. 17.92%, *P* = 3.52e-44) and Ery (22.7% vs. 25.8%, *P* = 1.43e-05) (Fig. S1E). Strikingly, IFN signaling-enriched HSC-2, Mono-1, and Ery-4 clusters were selectively depleted in the KO sample (Figs. 1C, D and S1F). Pseudo-bulk RNA-seq analysis revealed that loss of these three subsets attenuated IFN and inflammatory response in KO LKs (Fig. S1G, H). Ly6a (Sca1), an IFN-responsive gene and canonical surface marker for HSPCs [34, 35], displayed peak expression in WT HSC-2 populations, but exhibited consistently reduced expression in many KO clusters compared to WT counterparts (Fig. S1I). This transcriptional pattern suggests that the observed attenuation of IFN signaling in KO-LKs appears not merely attributable to the absence of HSC-2/Mono-1/Ery-4 subsets, but may stem from fundamental transcriptional dysregulation following *Setd2* deletion. Furthermore, the differentially expressed genes (DEGs) analysis revealed pan-repression of immune genes in each KO subgroup relative to WT (Fig. 1E). This was corroborated by hallmark Gene set enrichment analysis (GSEA), which showed impaired IFN and inflammatory pathways in the KO subgroup (Fig. 1F). Unexpectedly, our findings indicated that *Setd2* acts as a critical regulator of homeostatic inflammatory tonus in murine HSPCs.

### *Setd2* deficiency impairs IFN signaling in murine HSPCs at both steady and stimulated states

**Fig. 2:**
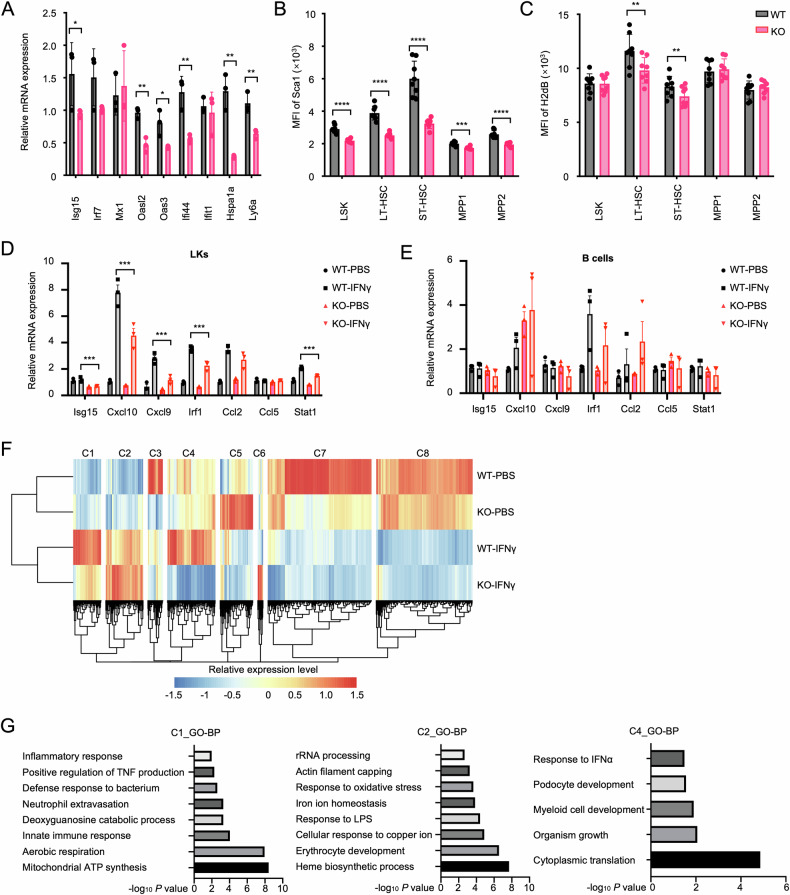
*Setd2* deficiency impairs IFN signaling networks in HSPCs. **A** RT-qPCR analysis of inflammatory gene expression in WT and KO LKs. Data normalized to *Gapdh* and presented as relative expression (2^−ΔΔCt^). *n* = 3. **B**, **C** Mean fluorescence intensity (MFI) detection of Ly6a (**B**) and H2dB (**C**) in HSPCs from WT and KO mice. LT-HSC, CD150^+^CD48^−^-LSKs; ST-HSC, CD150^−^CD48^−^-LSKs; MPP1, CD150^+^CD48^+^-LSKs; MPP2, CD150^−^CD48^+^-LSKs. WT, *n* = 8; KO, *n* = 7. **D**, **E** RT-qPCR analysis of IFN-target mRNA levels in WT/KO LKs (**D**) and B cells (**E**) after 30 min stimulation with IFNγ (1 ng/mL). *n* = 3. **F** Heatmap of clustering analysis of DEGs identified in pairwise comparisons between WT and KO LSKs after 24 hours of PBS or IFNγ (10 μg) treatment. **G** Bar chart showing top GO: biological process (GO-BP) terms for marker genes of cluster C1, C2, and C4.

To further support the decreased inflammatory state of KO-HSPCs, we conducted expression analysis of key IFN-target genes in KO-LKs. The transcriptional level of *Isg15*, *Oasl2*, *Oas3*, *Ifi44*, and *Ly6a* was reduced (Fig. 2A), with corresponding decreases in protein level of Sca1 and H2dB (major histocompatibility complex class I (MHC-I)) (Fig. 2B, C). Comparative analysis revealed that the compromised baseline IFN activity in KO-LKs further attenuated the induction of IFN-stimulated genes (ISGs), including *Cxcl10* and *Irf1*, following in vitro stimulation with 1 ng/mL IFNγ (Fig. 2D). Notably, splenic B cells from both genotypes maintained comparable ISG induction profiles under identical stimulation conditions (Fig. 2E). This cell type-specific deficiency in IFN responsiveness provides compelling evidence that *Setd2* exerts a critical regulatory function in HSPCs during IFN signaling cascades. Next, we analyzed the in vivo hematopoietic phenotype (Fig. S2A, illustrating the main gating strategy) before and after high-dose IFNγ treatment (24 h, 10 μg). Consistent with prior reports [36], high-dose IFNγ increased HSPC frequency (Fig. S2B), promoted cell cycle entry (Fig. S2C), and selectively modified downstream lineage composition relative to PBS controls (Fig. S2D–G), but no significant genotype-specific differences were observed post-stimulation. However, compared with that in the WT control, Sca1 intensity remained lower in KO long-term HSCs (LT-HSCs) post-exposure (Fig. S2H). Bulk RNA-seq analysis was then performed on BM LSKs under high-dose IFNγ stimulation. Cluster analysis of DEGs yielded eight distinct clusters (C1–C8) (Fig. 2F). Steady-state *Setd2* deficiency caused no changes in C1/C2/C4, but post-IFNγ responses diverged: WT showed robust C1/C2/C4 upregulation versus KO’s attenuated C1 response, exaggerated C2 activation, and C4 downregulation. C1/C4 genes linked to immune response and mitochondrial respiration, indicating that *Setd2* deficiency compromises the baseline inflammation response (Fig. 2G). The genes within C2 were predominantly enriched in heme biosynthesis and the metal ion metabolism process (Fig. 2G). Notably, C7 and C8, which were downregulated by *Setd2* deletion at steady state, were further downregulated to levels comparable to the WT group post-stimulation (Fig. 2F), indicating that the genes downregulated by *Setd2* deficiency at steady state were the important components of IFNγ-mediated gene repression [37]. Taken together, these findings demonstrate a bona fide IFN signaling defect in KO-HSPCs.

### Setd2 regulates the basal inflammatory signals in part by modulating chromatin accessibility and enhancer activity

To investigate whether extrinsic or intrinsic immune factors are involved in the regulation of immune signaling in *Setd2*-KO HSPCs, we analyzed cytokine expression in peripheral blood (PB) serum of WT and KO mice, but no genotype-specific differences were observed (Fig. S3A), suggesting that Setd2 is dispensable in cytokine-receptor binding-triggered signal transduction cascades in HSPCs. Previous studies have demonstrated that SETD2 can modulate the hepatic antiviral response by catalyzing the methylation of STAT1 [31]. Thus, we detected the phosphorylation level of Stat1 and Stat3 in WT- and KO-LKs under 100 ng/ml IFNγ exposure. However, IFNγ-stimulated Stat1/3 activation remained intact in KO LKs (Fig. S3B), indicating that Setd2 may not modulate immune signaling in HSPCs through the Stat1/3 pathway.

**Fig. 3:**
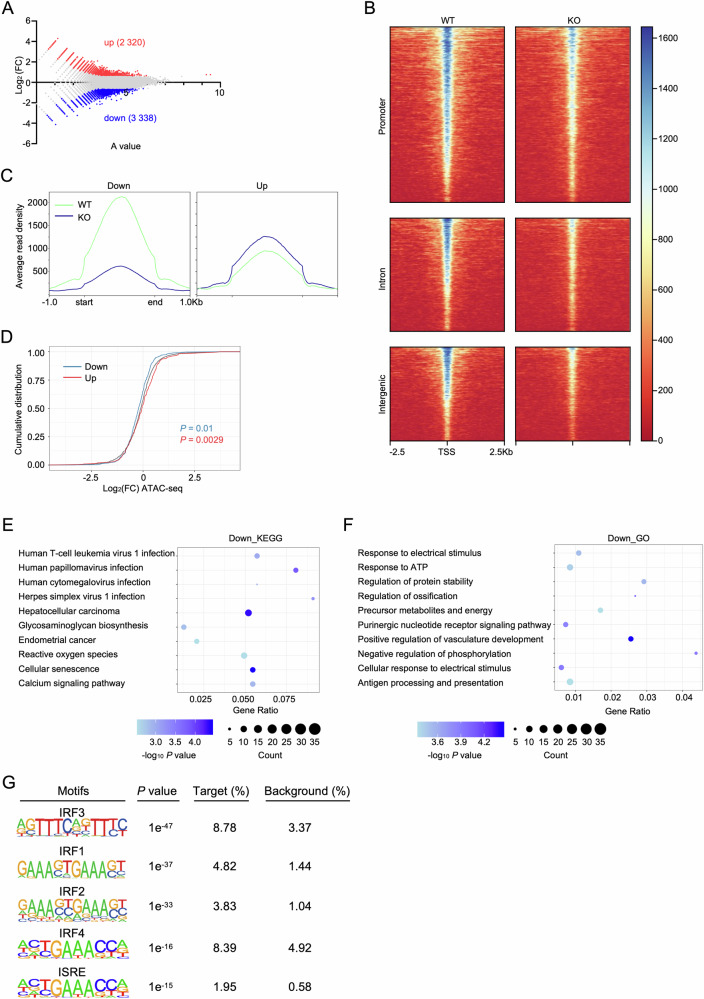
*Setd2* deficiency reduces chromatin accessibility at immunoinflammatory signaling-related genes. **A** Scatter plot of ATAC-seq peak accessibility changes in KO versus WT LSKs. Significantly altered peaks (*P* value < 0.05 and | log_2_ (FC) | ≥ 0.585) are labeled: upregulated (red), downregulated (blue). Gray dots: non-significant peaks. *A* value = log_2_ (A1 × B1)/2, where A1 and B1 represent the read densities in the KO and WT samples, respectively. **B** Heatmaps of accessible ATAC-seq peaks (±2.5-Kb window), grouped by genomic localization (promoter, intron, intergenic). Rows: individual peaks (Z-score normalized read density; red: low, blue: high accessibility). Peaks sorted by mean accessibility. **C** Average ATAC-seq signal profiles across all accessible genomic peaks in WT and KO LSKs. Metapeaks generated by aggregating peaks centered at summits (±1 kb). Signals normalized as reads per million (RPM). **D** Cumulative distribution function (CDF) graph of log_2_ (FC) for ATAC-seq peaks in KO versus WT LSKs. All peaks (gray), peaks linked to significantly upregulated (red) or downregulated (blue) genes are shown. *P* values calculated by the Kolmogorov–Smirnov test. **E**, **F** Functional enrichment analysis of genes linked to chromatin accessibility losses in KO LSKs. Bubble plots representing enriched **E** KEGG pathways and **F** GO-BP terms. Bubble color: enrichment significance (−log₁₀ *P* value); Size: number of core enriched genes (FDR < 0.05). **G** Top enriched TF binding motifs and predicted TFs in regions with decreased chromatin accessibility in KO LSKs. Identified via Homer motif analysis of significantly lost ATAC-seq peaks (FDR < 0.05, | log_2_ (FC) | ≥ 0.585).

Given that *Setd2* is an essential epigenetic regulator that can influence chromatin accessibility and alter the expression of downstream genes [38], we then employed Assay for Transposase-Accessible Chromatin with High Throughput Sequencing (ATAC-seq) to analyze chromatin accessibility profile of LSKs from WT and KO-BM. 2 320 open and 3 338 closed regions were uncovered in KO samples compared to WT control (*P* value < 0.05 and | log_2_ (fold change) (FC) | ≥ 0.585) (Fig. 3A), and the intensity of these peaks in KO genome was weaker than that in WT (Fig. 3B). Notably, the greater intensity loss in closed peaks versus gains in open regions (Figs. 3C and S3C) suggested *Setd2* deficiency tended to promote chromatin compaction. We then integrated this ATAC-seq data with the RNA-seq data on LSKs we published previously [23], showing accessibility-transcription coupling: upregulated genes associated with open chromatin, downregulated genes with closed domains (Fig. 3D). Kyoto Encyclopedia of Genes and Genomes (KEGG) and Gene Ontology (GO) analysis showed that closed regions enriched for antiviral/antigen presentation genes (Fig. 3E, F), while open regions associated with protein processing, oxidative stress, and myeloid cell differentiation (Fig. S3D, E). To identify the key transcription factors (TFs) responsible for the downregulation of inflammatory-related genes following *Setd2* deficiency, a motif analysis was performed. Motifs for IFN regulatory factor (IRF) family were highly enriched in closed chromatin regions (Fig. 3G), along with motifs for HSPC-related TFs (e.g., *Ets*, *Pu.1*, and *Runx1*) (Fig. S3F). Conversely, open chromatin harbored erythroid TF motifs (*Gata1/2*, *Scl*) (Fig. S3G), aligning with our previous finding that *Setd2* deficiency promotes erythroid differentiation [23].

**Fig. 4:**
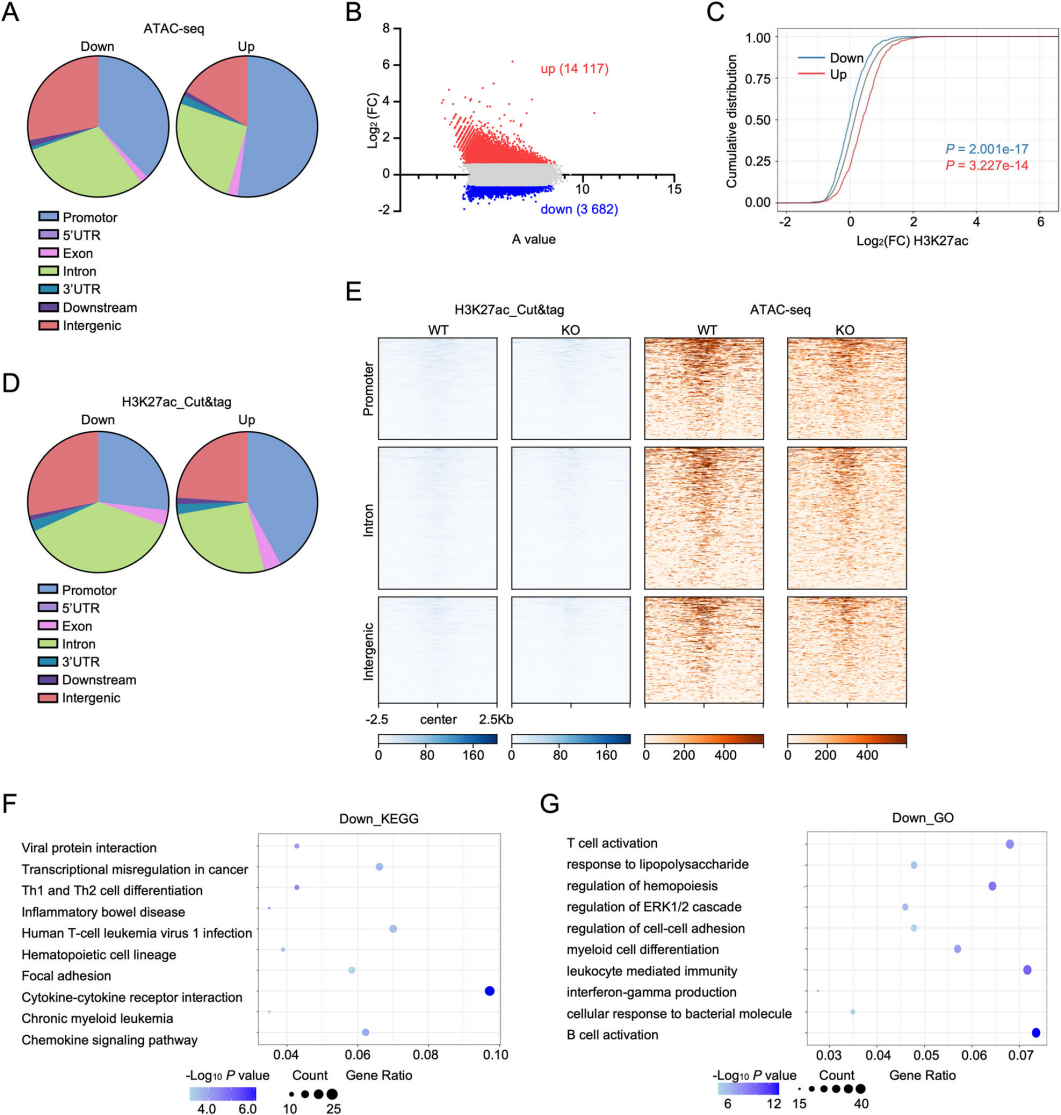
*Setd2* deficiency reduces enhancer activity at immunoinflammatory signaling-related genes. **A** Genomic distribution of differentially accessible ATAC-seq peaks in KO vs. WT LSKs. Proportions indicate preferential loss of accessible chromatin at intergenic and intronic regions. **B** Scatter plot of altered H3K27ac modification peaks in KO vs. WT LSKs. Significantly altered peaks (*P* value < 0.05 and | log_2_ (FC)| ≥ 0.585) are labeled: upregulated (red), downregulated (blue). Gray dots: non-significant peaks. **C** CDF graph of log_2_ (FC) for H3K27ac peaks in KO vs. WT LSKs. All peaks (gray), peaks linked to upregulated (red) or downregulated (blue) genes are shown. *P* values calculated by the Kolmogorov–Smirnov test. **D** Genomic distribution of differentially modified H3K27ac peaks in KO vs. WT LSKs. Proportions indicate preferentially deactivated enhancers at intergenic and intronic regions. **E** Paired heatmaps showing decreased H3K27ac modification peaks (left) and corresponding ATAC-seq accessibility (right) at identical genomic loci (±2.5-Kb window). Rows: individual genomic regions. **F**, **G** Bubble plots representing enriched (**F**) KEGG pathways and (**G**) GO-BP terms for genes associated with decreased H3K27ac peaks. Bubble color: enrichment significance (−log₁₀ *P* value); size: number of core enriched genes (FDR < 0.05).

Consistent with prior reports [39], ATAC-seq peaks localize predominantly to promoter, intronic, and intergenic regions. *Setd2* deficiency preferentially modified peak distribution by increasing promoter-associated peaks while reducing intronic/intergenic signals (Fig. S4A). Differential analysis of peak distribution further revealed that accessible chromatin gains concentrated in promoters, whereas losses localized to gene bodies (Fig. 4A). Since Setd2-catalyzed H3K36 trimethylation occurs predominantly in gene body regions, we hypothesized that this enzyme regulates transcription through enhancer modulation. Next, to compare enhancer activity between WT and KO LSKs, we performed a Cleavage Under Targets & Tagmentation (Cut&Tag) assay against H3K27ac. KO-LSKs exhibited enhancer hyperactivity (14 117 gained vs. 3 682 lost regions, *P* value < 0.05 and | log_2_ (FC) | ≥ 0.585) (Figs. 4B and S4B), with enhancer activities positively correlated with transcriptional output (Fig. 4C). Interestingly, deactivated enhancers clustered in gene body regions while activated elements populated in promoter regions (Fig. 4D). Subsequently, we integrated this Cut&Tag data with the ATAC-seq data above, and found that in *Setd2*-KO LSKs, the regions with decreased enhancer activity showed reduced chromatin accessibility (Fig. 4E). In line with closed peaks in ATAC-seq, KEGG and GO analysis of enhancer-silenced loci induced by *Setd2* deficiency showed profound enrichment of immune and inflammatory pathways, including those that regulate IFNγ production (Fig. 4F, G). Conversely, enhancers with increased activity are associated with genes involved in synapse formation, calcium signaling, and Wnt signaling (Fig. S4C, D). Taken together, these data suggest that *Setd2* deficiency disrupts the basal inflammatory signals in HSPCs in part by downregulating chromatin accessibility and enhancer activity.

### *Setd2* deficiency compromises the repopulation and differentiation activity of Sca1^high^-LSKs

**Fig. 5:**
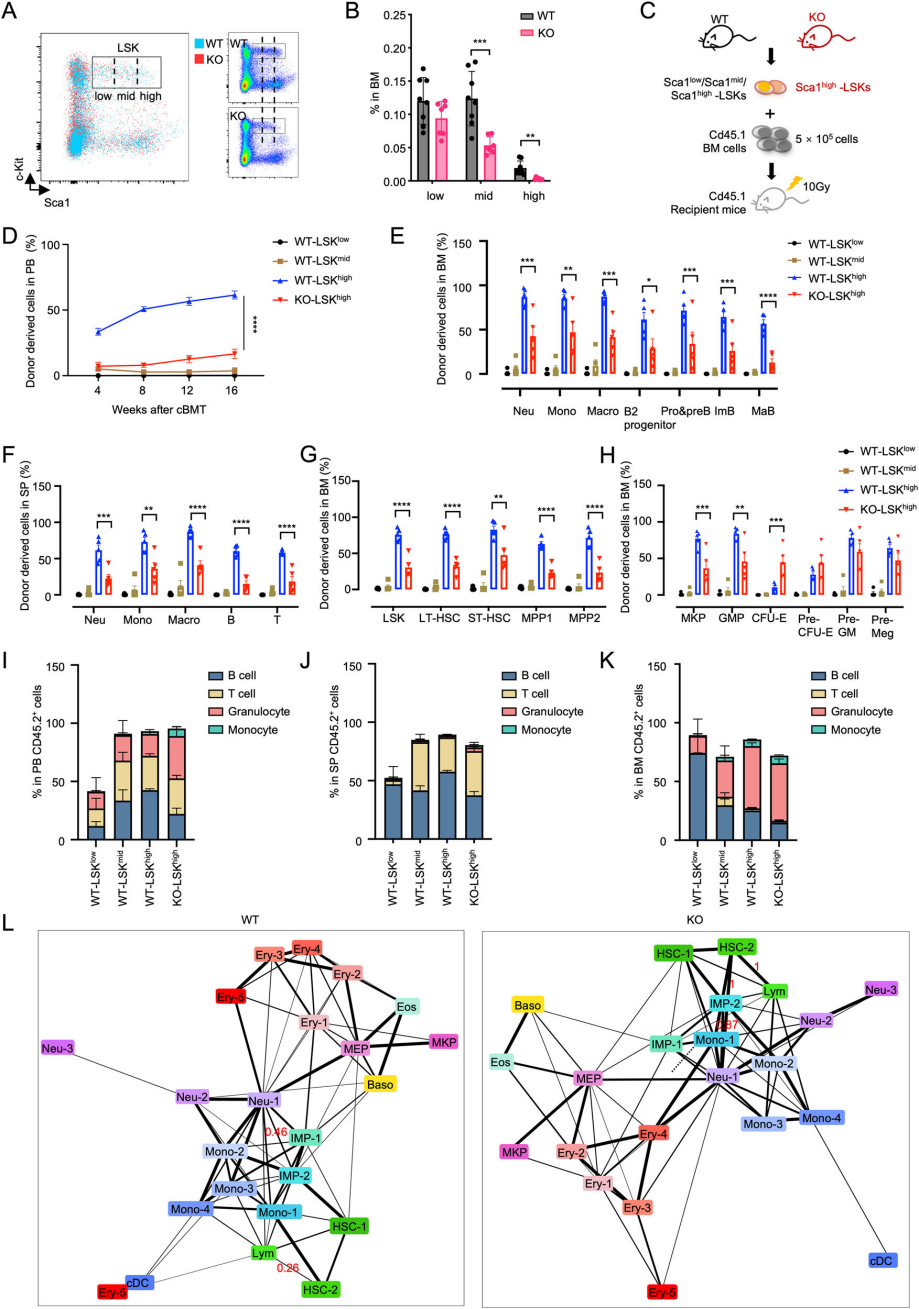
*Setd2* deficiency compromises repopulation and differentiation of Sca1^high^-LSKs. **A** Representative flow cytometry plot of gating Sca1^low^, Sca1^mid^, and Sca1^high^-LSKs based on Sca1 fluorescence intensity. **B** Frequency of Sca1^low^, Sca1^mid^, and Sca1^high^-LSKs in total BM. **C** Schematic of cBMT using LSK subsets from Cd45.2^+^ WT (Sca1^low^, Sca1^mid^, and Sca1^high^-LSKs) and KO (Sca1^high^-LSKs) mice into Cd45.1^+^ recipients. **D** Long-term donor-derived (Cd45.2^+^) cell frequency in PB of cBMT mice. *n* = 5. **E**–**K** Flow cytometry analysis of PB, spleen (SP), and BM from recipient mice 16 weeks post-transplantation. *n* = 5. **E** Donor-derived cell frequency in BM granulocyte-monocyte and B-cell lineage subsets. **F** Donor-derived cell frequency in spleen granulocyte-monocyte and lymphoid subsets. **G** Donor-derived cell frequency in LSKs and subsets. **H** Donor-derived cell frequency in hematopoietic progenitor subsets. **I–K** Frequency of B cells, T cells, granulocytes, and monocytes within donor-derived cells from PB (**I**), spleen (**J**), and BM (**K**). **L** PAGA prediction of developmental trajectories in WT (left) and KO (right) LKs. Line thickness reflects connectivity certainty between clusters.

It has been shown that immunoinflammatory signals (e.g., IFN) are essential for hematopoietic development (e.g., fetal HSC emergence and maturation, fetal-to-adult hematopoietic transition, and perinatal expansion of hematopoietic progenitor cells (HPCs) [2, 4, 40, 41]). To investigate the potential impact of insufficient immunoinflammatory signals on HSPC composition in *Setd2*-KO mice, we analyzed the frequency of immunophenotypic-HSPCs in the BM and showed that multipotent progenitor cells (MPPs) were significantly reduced in the KO group compared to the WT control (Fig. S5A). In addition, the population with high expression of Sca1 in KO-LSKs was notably reduced (Fig. 5A). Based on Sca1 expression levels, LSKs were artificially divided into Sca1^low^, Sca1^mid^, and Sca1^high^ subsets, with a significant reduction in Sca1^mid^ subset and a distinct absence of the Sca1^high^ subset in KO, relative to WT-BM (Fig. 5B). To exclude poly (I: C)-induction artifacts, we employed vav-Cre-mediated *Setd2* knockout, which efficiently deletes the floxed gene by embryonic day 13.5–14.5 [42]. As anticipated, the Sca1^high^-LSKs were also reduced in BM from adult vav-Cre *Setd2*-deficient mice (Fig. S5B, C), with the expression intensity of Sca1 being significantly reduced compared to WT LSKs (Fig. S5D). These results identify Sca1^high^-LSKs depletion as an intrinsic consequence of *Setd2* loss.

The reduction of Sca1^high^-LSKs mirrored the loss of HSC-2 subset (highly expressed *Ly6a*) in KO single-cell data. We therefore assessed the impact of *Setd2* deficiency on three Sca1-marked LSK subpopulations using serial colony-forming unit (CFU) assays, a method for evaluating the proliferation and differentiation potential of HSPCs in vitro. While primary CFU formation was comparable between KO and WT across subsets, secondary replating revealed genotype-specific differences: KO-Sca1^low^ and Sca1^high^ exhibited enhanced clonogenicity versus WT, whereas the KO-Sca1^mid^ was impaired (Fig. S5E). Intriguingly, this enhanced CFU potential in Sca1^low^ and Sca1^high^ subsets appeared inconsistent with our prior findings demonstrating that *Setd2* deficiency impairs the self-renewal capacity of HSCs in vivo. Given this apparent contradiction, we posited that altered differentiation kinetics, rather than preserved stemness characteristics, may underlie the observed CFU augmentation. Substantiating this hypothesis, *Setd2* deficiency enhanced macrophage progenitor cells (M) clonogenic potential of Sca1^high^ subset during serial replating (S5F).

Previous studies demonstrate that Sca1 intensity positively correlates with HSC repopulation capacity [43]. Accordingly, the Sca1^low^ subsets were biased toward MPPs commitment (with increased MPPs frequencies relative to other subsets), while Sca1^high^-LSKs maintained LT-HSC/short-term HSC (ST-HSC) predominance, with Sca1^mid^-LSKs showing intermediate output potential (Fig. S5G). However, WT Sca1^high^-LSKs exhibited weaker serial CFU capacity than Sca1^low/mid^ subsets in our assays (Fig. S5E). To resolve this discrepancy, we assessed Sca1 subpopulation reconstitution capacity in vivo by transplantation, focusing on *Setd2* deficiency effects in Sca1^high^-LSKs (Fig. 5C). Sca1 expression levels showed direct correlation with regenerative potential: WT Sca1^high^-LSKs generated > 50% of PB cells versus < 3% from Sca1^mid^-LSKs, while Sca1^low^-LSKs exhibited negligible reconstitution (Fig. 5D). *Setd2* deficiency markedly impaired Sca1^high^-LSKs repopulation activity (Fig. 5D). Comparative analysis revealed superior BM and spleen lineage reconstitution by WT Sca1^high^-LSKs versus Sca1^mid/low^ subsets, with *Setd2* loss compromising this capacity in Sca1^high^-LSKs (Fig. 5E, F). Similarly, HSPC-level reconstitution followed Sca1 expression gradients, while *Setd2* loss restricted repopulation at the HSPC level (Fig. 5G, H), demonstrating hierarchical functional heterogeneity in HSC fate determination. Based on BM transplantation (BMT) results, we believe that the self-renewal defect caused by *Setd2* deficiency is partly attributed to the lack of functional HSC in Sca1^high^-LSKs.

Next, we interrogated the role of *Setd2* deletion in functional-HSC differentiation. Functional assessment of Sca1^high^-LSK-derived lineages across hematopoietic compartments confirmed compromised B-cell differentiation potential in KO populations (PB, spleen, BM) (Fig. 5I–K). We employed partition-based graph abstraction (PAGA) to analyze the differentiation disparities between WT- and KO-LKs [44]. Comparative analysis of WT- and KO-LK populations revealed enhanced differentiation potential from HSC-2 to Lym (confidence value (CV) of HSC-2 to Lym: KO vs. WT, 1 vs. 0.264) and Neu-1 (CV: 1 vs. 0) in KO specimens (Fig. 5L). While HSC-1 maintained equivalent Lym differentiation capacity in both genotypes (CV: 0.4 vs. 0.4), HSC-2 depletion in KO samples (Fig. S1E) primarily drove observed lymphoid reduction. Neu-1 populations remained stable through compensatory differentiation from multiple precursors, such as IMP-2 (CV: 0.87 vs 0.46). These findings establish that *Setd2* deficiency impairs both Sca1^high^-LSKs maintenance and B lymphoid commitment.

### *Setd2* deficiency confers resistance to HSPC exhaustion by long-term IFNγ treatment

**Fig. 6:**
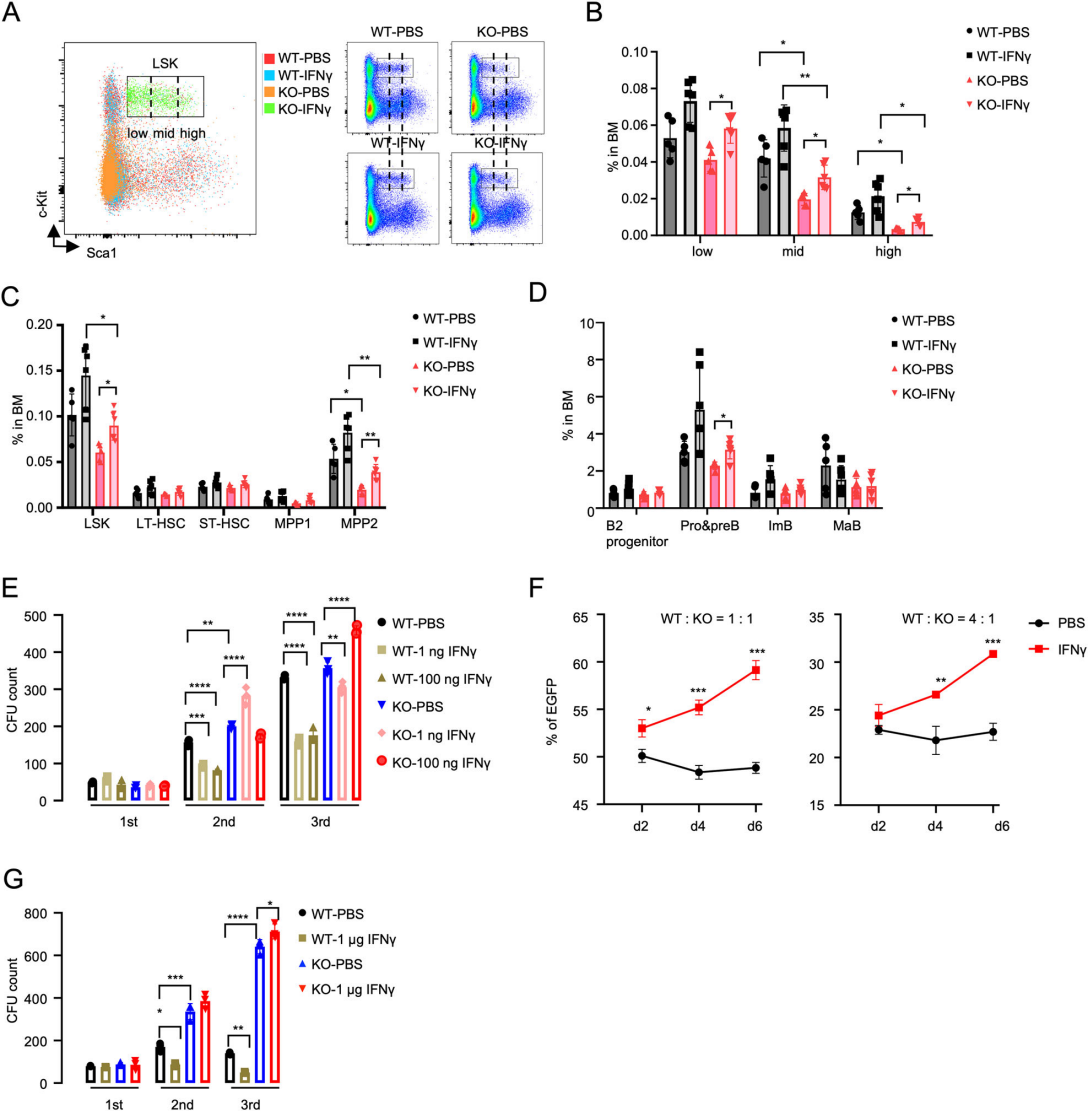
*Setd2* deficiency impedes IFNγ-induced HSPC exhaustion. **A**–**D** Flow cytometry analysis of BM from WT (PBS, *n* = 5; IFNγ, *n* = 6) and KO (PBS, *n* = 5; IFNγ, *n* = 6) mice after 3-week IFNγ treatment (1 μg/day). **A** Representative LSK subsets gating. **B** Frequency of Sca1^low^, Sca1^mid^, and Sca1^high^-LSKs in total BM. **C** Frequency of LSKs and subsets in total BM. **D** Frequency of B-cell lineage subsets in total BM. **E** Serial CFU assays of WT and KO LSKs cultured with PBS, 1 ng/mL IFNγ, and 100 ng/mL IFNγ. *n* = 3. **F** Cell growth analysis evaluating the impact of IFNγ (50 ng/mL) on BM LKs (left, WT: KO = 1: 1; right, WT: KO = 4: 1). The proportion of KO cells (EGFP positive) is calculated at different time points after treatment. *n* = 3. **G** Serial CFU assays of BM cells from WT and KO mice after 3-week PBS/IFNγ treatment (1 μg/day).

Given that *Setd2* loss impairs self-renewal, reduces Sca1^high^-LSKs, and attenuates IFN signaling across HSPC subpopulations, we tested the rescue effects of IFNγ supplementation. Low-dose IFNγ (1 μg/day for 3 weeks) increased all Sca1 subsets in KO mice compared with PBS controls (Fig. 6A, B), which was mainly reflected by selective expansion of MPP2 (Fig. 6C). Notably, ANOVA analysis showed no significant difference in IFNγ-induced Sca1 intensity between WT and KO; however, within the KO group, IFNγ increased Sca1 intensity in LT-HSCs, ST-HSCs, and MPP2 (Fig. S6A, *P* < 0.05 analyzed by Student’s *t* test). IFNγ partially restored KO B-cell differentiation through pro/pre-B-cell expansion in KO-BM (Fig. 6D) but spared other lineages in both genotypes (Fig. S6B–D). Competitive BMT (cBMT) with continuous low-dose IFNγ supplementation (1 μg/day for 3 weeks) failed to rescue the reconstitution defect in KO-HSPCs (Fig. S6E–G), likely due to IFNγ‘s preferential expansion of MPP2 over functional LT-HSCs.

Emerging evidence links chronic inflammation to hematopoietic dysfunction. Although IFNγ-induced HSPC exhaustion is well-documented [36], *Dnmt3a* deletion confers resistance to IFNγ stimulation while promoting clonal hematopoiesis and hematopoietic transformation [45]. Given that *Setd2*-deficient mice develop MDS-like disorder, which is an inflammation-driven hematological malignancy [46], we investigated the responses of *Setd2*-mutated HSPCs to IFNγ stimulation. Serial replating revealed IFNγ-enhanced CFU capacity in KO-LSKs versus inflammation-induced exhaustion in WT counterparts (Fig. 6E). Competitive co-cultures demonstrated significant proliferation advantages for KO-LKs under IFNγ stimulation (Fig. 6F). In vivo validation through 3-week IFNγ treatment showed diametrically opposed responses: while WT-BM cells exhibited functional impairment, KO-BM cells displayed enhanced replating capacity under inflammatory challenge (Fig. 6G). Collectively, *Setd2* deficiency establishes baseline inflammatory signaling defects that paradoxically confer resistance to hematopoietic stress through compensatory fitness adaptation.

## Discussion

HSPCs, as the progenitors of immune cells, constitutively express PRRs and MHC-II molecules, enabling them to produce cytokines at levels exceeding those of mature immune cells and to present antigens [47, 48]. However, mechanisms maintaining baseline inflammatory/immune signals in HSPCs remain unclear. While epigenetic regulators (e.g., *Dnmt3a*, *Kdm6b*, *Tet2*) suppress inflammatory signals [45, 49, 50], we identified the H3K36me3 methyltransferase *Setd2* as essential for sustaining baseline inflammatory signaling—particularly IFN activity—in HSPCs. To our knowledge, Setd2 was the first positively regulated epigenetic factor that has been identified to maintain the baseline inflammatory state of HSPCs.

Although constitutively expressing IFN-related genes, HSCs with IFN receptor deficiency maintain intact self-renewal capacity and differentiation potential [41]. However, we observed that *Setd2* deficiency attenuated IFN signaling, accompanied by the depletion of Sca1^high^-LSKs—a population exhibiting high IFN activity and specifically enriched with functional HSCs. To probe the relationship between IFN and HSC function in our system, we stimulated WT and KO mice with low-dose IFNγ in vivo. While IFNγ expanded KO Sca1^high^-LSKs (primarily MPP2), it failed to rescue their reconstitution defect in competitive transplants. This finding demonstrated that additional IFNγ failed to induce hematopoietic reconstitution due to the absence of functional Sca1^high^-HSCs. We therefore proposed that the self-renewal defect in *Setd2*-deficient HSCs likely stems from dual quantitative and functional impairments: 1) Insufficient IFN signaling during development resulted in impaired generation of an adequate functional-HSC pool (IFN activity-dependent); and 2) Post-developmental *Setd2* deficiency compromised the self-renewal capacity of established functional HSCs through other unidentified mechanisms (IFN activity-independent). We will address these two points in the future.

*Setd2* deficiency not only depleted the formation of Sca1^high^-LSKs with substantial IFN activity but also impaired IFN signaling across most existing HSPC subpopulations. It has been shown that epigenetic regulation, particularly chromatin accessibility remodeling, governs IFN pathway activation during fetal-to-adult hematopoietic transition [2]. Consistently, *Setd2* deficiency restricted chromatin accessibility and H3K27ac-marked enhancers at inflammation-associated genes in adult HSPCs. A critical unresolved question is whether the restricted chromatin accessibility of *Setd2*-deficient inflammatory genes reflects (1) failure to initiate accessibility during fetal-to-adult hematopoietic transition, or (2) secondary shutdown after HSPC maturation.

The IFN activity operates within a narrow range in vivo. Insufficient IFN activity leads to immunodeficiency and impaired antiviral defense, while excessive signaling predisposes to autoimmune disorders [51]. Hepatic *Setd2* deficiency has been shown to cause defective antiviral immune responses [31], consistent with clinical observations of *SETD2* mutations in immunodeficient patients [26]. Although *Setd2* deficiency-mediated B lymphopenia has been proposed as a potential mechanism for PID, our study not only supports this hypothesis but also provides novel insights from an HSPC perspective. We demonstrated that *Setd2* deficiency reduced both basal IFN activity in HSPCs and their responsiveness to exogenous IFNγ stimulation. Transcriptome analysis revealed impaired upregulation of several ISGs in IFNγ-treated KO-LSKs compared to WT counterparts. Intriguingly, our data also identified a subset of *Setd2*-regulated genes in LSKs that are intrinsically involved in IFN-mediated transcriptional repression. Beyond its direct effects on HSPCs, *Setd2* deficiency perturbs downstream differentiation, with reduced B-lineage commitment potentially attributable to insufficient IFN pathway activity. Notably, we observed concurrent depletion of three cell populations in KO mice: the HSC-2 subset along with Mono-1 and Ery-4 subpopulations, all exhibiting enriched IFN signatures. The absence of definitive surface markers for Mono-1 and Ery-4 subpopulations precluded their isolation and functional characterization using standard approaches like Sca1^high^-LSKs sorting. Previous studies have classified monocyte origins into G-mono (LPS-responsive) and M-mono (CpG-responsive) transcriptional subtypes, suggesting distinct roles in bacterial versus viral immunity [52]. The absence of Mono-1 may alter the G-mono/M-mono composition in KO mice, potentially compromising appropriate immune responses to specific pathogens. The human C4 cluster (an immune-erythroid subset) co-expresses core erythroid regulators and immune-effector genes, enabling immunomodulation [53]. Loss of Ery-4, which might align with the evolutionarily conserved human C4 cluster, suggests disrupted integration of erythroid maturation with stress-responsive immunity.

Although we did not use live viral infections in KO mice to replicate findings observed in liver-specific *Setd2*-deficient models [31], our study demonstrated that insufficient IFN signaling served as a critical mechanism enabling HSPCs to evade inflammation-induced exhaustion. This finding advanced our understanding of inflammation-driven clonal selection and malignant transformation. Chronic inflammation is well-documented to exert opposing regulatory effects on normal and mutant HSPCs: persistent inflammatory stimulation depletes normal HSPCs while promoting mutant clonal expansion and oncogenic progression [9]. Mechanistic studies have elucidated selective advantages in mutant HSPCs. For instance, *Tet2* deficiency activates IL-6 signaling but concurrently suppresses pro-apoptotic gene expression in HSPCs, conferring survival advantages under inflammatory stress [15, 54]. Similarly, *Dnmt3a*-deficient HSPCs exhibit hyperactivation of IFN signaling yet resist IFNγ-induced differentiation exhaustion due to impaired differentiation caused by *Dnmt3a* loss [45]. In *Asxl1*-mutant HSPCs, concurrent upregulation of pro-inflammatory and anti-inflammatory pathways raises the threshold for inflammatory tolerance, enabling these cells to withstand persistent inflammatory stimuli [17]. Our findings proposed an alternative tolerance mechanism: *Setd2*-deficient HSPCs attenuated baseline IFN signaling, thereby reducing pathway activation below the threshold required for inflammatory exhaustion upon IFN stimulation. This adaptation promotes survival under inflammatory stress. Collectively, our work establishes a critical link between *Setd2* deficiency, dysregulated inflammation-immune crosstalk, and the pathogenesis of malignancy.

## Materials and methods

### Mouse model

Setd2 ^fl/fl^ mice were generated as previously reported [23]. Mx1-Cre, vav-Cre, and CD45.1 mice were purchased from Shanghai Model Organisms Center (Shanghai, China). Setd2 ^fl/fl^ Mx1/vav-Cre^+/−^ mice were intercrossed with Setd2 ^fl/fl^ Mx1/vav-Cre^−/−^ mice to obtain Setd2 ^fl/fl^ Mx1/vav-Cre^+/−^ mice (KO) and Setd2 ^fl/fl^ Mx1/vav-Cre^−/−^ (WT) mice. The Mx1-Cre transgene was induced in 4-week-old mice using poly(I: C) (tlrl-pic-5, InvivoGen, 5 mg/kg) administered i.p. every other day, three times. All mouse experiments were conducted in accordance with the animal care standards (DLA-MP-IACUC.06) from the Institutional Animal Care and Use Committee (IACUC) of Shanghai Jiao Tong University School of Medicine (Shanghai, China).

### Flow cytometry and cell sorting

BM cells were harvested from WT and KO mice at 3 weeks post poly (I: C) treatment. Cells were flushed from the femur and tibia using RPMI 1640 medium (LR1634, Bioagrio), suspended in phosphate buffer saline (PBS, ST476, Beyotime) supplemented with 1% fetal bovine serum (FBS, DCF-201-0500, Dcell biologics), and then stained with the indicated antibodies to detect HSPCs and downstream lineage mature cells. Flow cytometry was conducted on BD LSRFortessa™ X-20 (BD Biosciences), and data were analyzed using FlowJo software. LKs and LSKs were sorted from WT and KO-BM cells using BD FacsAria™ III (BD Biosciences). A list of antibodies used for flow cytometry analysis and cell sorting is provided in Table S2.

### BM transplantation assay

BM cells were isolated from WT and KO donor mice at 3 weeks post poly (I: C) treatment. For cBMT, BM cells from donor mice (CD45.2) of different genotypes and competitor mice (CD45.1) were mixed at a 1: 1 ratio (5 × 10^5^ cells each) and injected into the lateral tail vein of 8-week-old CD45.1 recipient mice that had been exposed to a lethal dose of 10 Gy irradiation. The recipient mice were administered a low dose of IFNγ (i.p., 1 μg/day, C746, Novoprotein) for three weeks and were subsequently sacrificed for phenotype analysis. For LSK subsets (Sca1^low^, Sca1^mid^, and Sca1^high^), 2000 cells each sorted from donor mice (CD45.2) were mixed with BM cells (5 × 10^5^ cells) from competitor mice (CD45.1) and injected into CD45.1 lethally irradiated recipient mice. PB was collected from recipient mice every 4 weeks, and the mice were sacrificed for phenotype analysis 16 weeks post-transplantation.

### Methyl cellulose assays and liquid culture

BM cells (1 × 10^4^ cells) or 100 LSKs (stimulated with PBS, 1 ng/mL or 100 ng/mL IFNγ) or 100 LSK subsets (Sca1^low^, Sca1^mid^, and Sca1^high^-LSKs), isolated from WT and KO mice, were plated in MethoCult™ GF M3434 medium (#03434, Stem Cell Technologies). After incubation at 37 °C in a 5% CO2 incubator for 7 days, colonies were classified and counted based on morphology as granulocyte (G), M, granulocyte-macrophage (GM), erythroid (E), and multi-lineage (GEMM) progenitor cells. For serial replating, 1 × 10^4^ colonies harvested from the prior generation’s CFU were replated into fresh M3434 medium. Colonies were counted after an additional 7 days of incubation.

To compare the ability of inflammatory resistance in WT and KO mice, LKs were sorted and cultured in RPMI 1640 medium supplemented with 10% FBS, 20 ng/mL SCF (C775, Novoprotein), 20 ng/mL Flt-3L (CC19, Novoprotein), penicillin-streptomycin (15070-063, Gibco), plasmocin (ant-mpp, InvivoGen), and 50 ng/mL IFNγ. WT and KO cells were mixed at the ratios of 1: 1 and 4: 1, and the change of cell ratios was detected through flow cytometry (cells from KO mice expressed EGFP [23], and the percentage of EGFP^+^ cells can indicate the ratio of KO cells).

### PB analysis

PB was collected retro-orbitally at the indicated time points from primary and transplanted mice. The frequencies of monocytes, neutrophils, and lymphocytes in PB were analyzed on BD LSRFortessa™ X-20 after the removal of RBCs using ammonium-chloride-potassium lysis buffer.

### Western blot analysis

BM cells were isolated, and LKs were sorted from WT and KO mice at 3 weeks post poly (I: C) treatment. Subsequently, LKs were treated with 100 ng/mL IFNγ at 37 °C in a 5% CO2 incubator for 20 minutes. Whole-cell protein lysates were prepared using 1× SDS sample buffer (Beyotime, P0015), and Western blot analysis was performed using antibodies against total Stat1 (CST, 14994), phosphorylated Stat1 (Tyr701) (CST, 7649), total Stat3 (CST, 9139), phosphorylated Stat3 (Tyr705) (CST, 9145), H3K36me3 (abcam, ab9050), H3 (CST, 4499 s), and β-actin (Yeason, 30101ES60), respectively.

### Cytokine detection in PB serum

PB serum was collected from WT and KO mice at 3 weeks post poly (I: C) treatment. Cytokines were detected using a LEGENDplex^TM^ Mouse Inflammation Cytokine Panel array kit (BioLegend, 740165) following the manufacturer’s guidelines. Data analysis was performed on the Qognit platform at https://legendplex.qognit.com/user/login/.

### Cell cycle analysis

BM cells were harvested and stained with antibodies to identify HSPCs from WT and KO mice before and after IFNγ stimulation (i.p., 10 μg). Cells were fixed with BD Cytofix/Cytoperm™ Fixation (BD Biosciences, 554722) for 1 hour at 4 °C, then washed twice with BD Perm/Wash™ buffer (BD Biosciences, 554723). Subsequently, cells were stained with anti-Ki67 antibodies (Biolegend, 652404) for 30 minutes at 4 °C and Hoechst 33342 (Life Technologies, H3570) for 20 minutes at 4 °C. The flow cytometry analysis was performed immediately afterward.

### IFNγ in vitro stimulation

Splenic B cells were positively selected for CD19^+^ cells from WT and KO mice using magnetic-activated cell sorting (MACS, #76447, BioLegend) according to the manufacturer’s instructions. LKs were sorted from WT and KO-BM cells. Subsequently, cells were treated with IFNγ (1 ng/mL, C746, Novoprotein) at 37 °C in a 5% CO_2_ incubator for 30 mins. Total RNA was immediately isolated using TRIzol™ reagent (#15596018, Ambion by Life Technologies) according to the manufacturer’s protocol.

### Real-time quantitative PCR (RT-qPCR)

Total RNA was reverse transcribed using the High-Capacity cDNA Reverse Transcription Kit (#4368814, Thermo Fisher Scientific). RT-qPCR was conducted using ChamQ SYBR qPCR Master Mix (Low ROX Premixed) (#Q331-02, Vazyme) on LightCycler® 480 II (Roche). Data were normalized to *Gapdh* and presented as relative expression levels (2^−ΔΔCt^). A list of primers is provided in Table S2.

### Bulk RNA-seq

Total RNAs from sorted LSKs from WT and KO mice before and after IFNγ stimulation (i.p., 10 μg) were extracted using Trizol (Invitrogen, 15596018). The RNAs were quantified using a Qubit®3.0 Fluorometer (Life Technologies) and the Nanodrop One spectrophotometer (Thermo Fisher Scientific). Subsequently, RNA samples were fragmented and subjected to first-strand and second-strand cDNA synthetization. cDNA was ligated with sequencing adapters following the standard Illumina NovaSeq 6000 protocol. The sequencing reads were then mapped to the mouse reference genome (GRCm38.102) using Hisat2 (http://daehwankimlab.github.io/hisat2/, version: 2.1.0). Stringtie (http://ccb.jhu.edu/software/stringtie/index.shtml/, version: 2.0.0) was used to calculate fragments per kilobase of exon model per million mapped reads (FPKM) values, and edgeR (https://bioconductor.org/packages/release/bioc/html/edgeR.html/, version: 3.17) was employed to analyze DEGs based on FC ≥ 2 and *P* < 0.05.

### ScRNA-seq and statistical analysis

LKs were sorted from WT and KO mice at 3 weeks post poly (I: C) treatment. The BD Rhapsody system (BD Biosciences) was used to capture the transcriptomic profiles of single cells. Utilizing the BD Rhapsody single-cell whole-transcriptome amplification workflow, whole-transcriptome libraries were constructed. The library was sequenced on a NovaSeq6000 platform (Illumina) with a 150 bp paired-end run. To acquire the clean data, Fastp (https://github.com/OpenGene/fastp/, version: 0.20.1) was applied with default parameters to trim sequencing adapters and eliminate low-quality reads. The cleaned data were aligned to the reference mouse genome (Ensemble, version: 92) utilizing STAR with customized parameters to generate UMI counts. Cell barcodes with UMI < 500 or mitochondria UMI rate > 25% were excluded based on cell quality criteria. Cell normalization and regression based on the UMI count and the percentage of mitochondria rate were performed to scale the data, which was then analyzed using the Seurat package (https://satijalab.org/seurat/, version: 2.3.4). Principal component analysis was conducted with default parameters to reduce dimensionality. An unsupervised cell cluster was derived from the top principal components using a graph-based cluster method (resolution = 0.6). Marker genes were calculated using the FindAllMarkers function with the Wilcoxon Test, applying criteria including log_2_ (FC) > 0.25; minimum percentage (min.pct) > 0.25. For data visualization, a UMAP cell embedding was generated using the RunUMAP function. Pseudo-bulk expression profiles were computed for each cluster, and EdgeR was used to examine differential expression. Expression modules were recovered through hierarchical clustering of 733 DEGs detected in at least one of 22 clusters, revealing six modules (Table S3). Genes identified as markers were subjected to GSEA (https://www.gsea-msigdb.org/gsea/index.jsp/), utilizing gene sets from the Molecular Signature Database (MSigDB). The PAGA algorithm analysis was applied to visualize the lineage relationships among all identified cell subpopulations [44].

### GO and KEGG analysis

For GO enrichment analysis, the representative profiles of DEGs were analyzed using DAVID software (http://david.abcc.ncifcrf.gov/). The significant pathways of these DEGs were identified based on the KEGG database (https://www.genome.jp/kegg/). The threshold for significance was set using the *P* value and false discovery rate (FDR).

### ATAC-seq

LSKs were sorted from WT and KO mice at 3 weeks post poly (I: C) treatment. Approximately 5 × 10^4^ LSKs were then used for ATAC-seq DNA library construction with the TruePrep DNA Library Prep Kit V2 for Illumina (Vazyme, TD502), following the manufacturer’s guidelines. PCR was employed to amplify the transposed DNA fragments using primers with different barcodes. The library was sequenced on a NovaSeq 6000 (Illumina), and the data were aligned to the reference mm10 genome utilizing Bowtie2. MACS2 was utilized to perform peak calling. Homer, with default parameters, was applied to conduct motif calling. ChIPseeker was employed for peak annotation and to screen for genes associated with the binding site for functional enrichment analysis. MAnorm was utilized for sample difference analysis.

### Cut&Tag

LSKs were sorted from WT and KO mice at 3 weeks post poly (I: C) treatment. Approximately 1 × 10^5^ LSKs were then utilized for Cut&Tag DNA library construction with NovoNGS^®^ CUT&Tag^®^ 3.0 High-Sensitivity Kit for Illumina^®^ (Novoprotein, N259-YH01), adhering to the manufacturer’s guidelines. PCR amplification of the library was conducted using primers supplied with the kit. Post-PCR purification was carried out using NovoNGS^®^ DNA Clean Beads. The library was sequenced on a NovaSeq 6000 (Illumina), and the resulting data were aligned to the reference mm10 genome utilizing Bowtie2. Peak calling was performed using MACS2. ChIPseeker was employed for peak annotation and to screen for genes associated with binding site for functional enrichment analysis. MAnorm was utilized for sample difference analysis.

### Statistical analysis

An unpaired two-tailed Student’s *t* test was employed for two-group comparisons (e.g., WT vs. KO), and ANOVA was applied for experiments involving more than two groups (e.g., WT, KO, and treated groups). The Chi-square test used on bioinformatic data is described in the relevant figure legend. Differences between groups were considered statistically significant at *P* < 0.05 (*P < 0.05, **P < 0.01, ***P < 0.001, ****P < 0.0001). All statistical analyses were conducted using GraphPad Prism software and R software. Data are presented as means ± standard error of the mean (SEM).

## Supplementary information

Supplementary Materials

Table S1. AllMarkerGenes

Table S2. Flow cytometry antibodies and RT-qPCR primers

Table S3. DE_modules

Supplementary WB results

## Data Availability

All sequencing data included in this study are available in the National Omics Data Encyclopedia (NODE) (accession numbers: OEP00005691 and OEP00005707). All data generated or analyzed in this study are included in this published article.

## References

[1] King KY, Goodell MA. Inflammatory modulation of HSCs: viewing the HSC as a foundation for the immune response. Nat Rev Immunol. 2011;11:685–92. 21904387 10.1038/nri3062 PMC4154310

[2] Li Y, Kong W, Yang W, Patel RM, Casey EB, Okeyo-Owuor T, et al. Single-cell analysis of neonatal HSC ontogeny reveals gradual and uncoordinated transcriptional reprogramming that begins before birth. Cell Stem Cell. 2020;27:732–47.e7. 32822583 10.1016/j.stem.2020.08.001 PMC7655695

[3] Cai S, Li H, Tie R, Shan W, Luo Q, Wang S, et al. Nlrc3 signaling is indispensable for hematopoietic stem cell emergence via notch signaling in vertebrates. Nat Commun. 2024;15:226. 38172511 10.1038/s41467-023-44251-6 PMC10764762

[4] Espin-Palazon R, Stachura DL, Campbell CA, Garcia-Moreno D, Del Cid N, Kim AD, et al. Proinflammatory signaling regulates hematopoietic stem cell emergence. Cell. 2014;159:1070–85. 25416946 10.1016/j.cell.2014.10.031 PMC4243083

[5] Lefkopoulos S, Polyzou A, Derecka M, Bergo V, Clapes T, Cauchy P, et al. Repetitive elements trigger RIG-I-like receptor signaling that regulates the emergence of hematopoietic stem and progenitor cells. Immunity. 2020;53:934–51 e9. 33159854 10.1016/j.immuni.2020.10.007

[6] Pietras EM. Inflammation: a key regulator of hematopoietic stem cell fate in health and disease. Blood. 2017;130:1693–8. 28874349 10.1182/blood-2017-06-780882 PMC5639485

[7] Barreyro L, Chlon TM, Starczynowski DT. Chronic immune response dysregulation in MDS pathogenesis. Blood. 2018;132:1553–60. 30104218 10.1182/blood-2018-03-784116 PMC6182269

[8] Stubbins RJ, Platzbecker U, Karsan A. Inflammation and myeloid malignancy: quenching the flame. Blood. 2022;140:1067–74. 35468199 10.1182/blood.2021015162

[9] Caiado F, Pietras EM, Manz MG Inflammation as a regulator of hematopoietic stem cell function in disease, aging, and clonal selection. J Exp Med. 2021;218:e20201541. 10.1084/jem.20201541 PMC8210622 34129016

[10] Collins A, Mitchell CA, Passegue E Inflammatory signaling regulates hematopoietic stem and progenitor cell development and homeostasis. J Exp Med. 2021;218:e20201545. 10.1084/jem.20201545 PMC8210624 34129018

[11] Lee SC, North K, Kim E, Jang E, Obeng E, Lu SX, et al. Synthetic lethal and convergent biological effects of cancer-associated spliceosomal gene mutations. Cancer Cell. 2018;34:225–41.e8. 30107174 10.1016/j.ccell.2018.07.003 PMC6373472

[12] Guillamot M, Ouazia D, Dolgalev I, Yeung ST, Kourtis N, Dai Y, et al. The E3 ubiquitin ligase SPOP controls resolution of systemic inflammation by triggering MYD88 degradation. Nat Immunol. 2019;20:1196–207. 31406379 10.1038/s41590-019-0454-6 PMC7376385

[13] Chen Z, Amro EM, Becker F, Holzer M, Rasa SMM, Njeru SN, et al. Cohesin-mediated NF-kappaB signaling limits hematopoietic stem cell self-renewal in aging and inflammation. J Exp Med. 2019;216:152–75. 30530755 10.1084/jem.20181505 PMC6314529

[14] Gao Y, Vasic R, Song Y, Teng R, Liu C, Gbyli R, et al. m(6)A modification prevents formation of endogenous double-stranded rnas and deleterious innate immune responses during hematopoietic development. Immunity. 2020;52:1007–21.e8. 32497523 10.1016/j.immuni.2020.05.003 PMC7408742

[15] Cai Z, Kotzin JJ, Ramdas B, Chen S, Nelanuthala S, Palam LR, et al. Inhibition of inflammatory signaling in Tet2 mutant preleukemic cells mitigates stress-induced abnormalities and clonal hematopoiesis. Cell Stem Cell. 2018;23:833–49.e5. 30526882 10.1016/j.stem.2018.10.013 PMC6317370

[16] Xie J, Sheng M, Rong S, Zhou D, Wang C, Wu W, et al. STING activation in TET2-mutated hematopoietic stem/progenitor cells contributes to the increased self-renewal and neoplastic transformation. Leukemia. 2023;37:2457–67. 37816954 10.1038/s41375-023-02055-z PMC10681905

[17] Avagyan S, Henninger JE, Mannherz WP, Mistry M, Yoon J, Yang S, et al. Resistance to inflammation underlies enhanced fitness in clonal hematopoiesis. Science. 2021;374:768–72. 34735227 10.1126/science.aba9304

[18] Shi Z, Liu J, Zhao Y, Yang L, Cai Y, Zhang P, et al. ASXL1 mutations accelerate bone marrow fibrosis via EGR1-TNFA axis-mediated neoplastic fibrocyte generation in myeloproliferative neoplasms. Haematologica. 2023;108:1359–73. 36005555 10.3324/haematol.2021.280320 PMC10153516

[19] Sun XJ, Wei J, Wu XY, Hu M, Wang L, Wang HH, et al. Identification and characterization of a novel human histone H3 lysine 36-specific methyltransferase. J Biol Chem. 2005;280:35261–71. 16118227 10.1074/jbc.M504012200

[20] Hu M, Sun XJ, Zhang YL, Kuang Y, Hu CQ, Wu WL, et al. Histone H3 lysine 36 methyltransferase Hypb/Setd2 is required for embryonic vascular remodeling. Proc Natl Acad Sci USA. 2010;107:2956–61. 20133625 10.1073/pnas.0915033107 PMC2840328

[21] Zhang Y, Fang Y, Tang Y, Han S, Jia J, Wan X, et al. SMYD5 catalyzes histone H3 lysine 36 trimethylation at promoters. Nat Commun. 2022;13:3190. 35680905 10.1038/s41467-022-30940-1 PMC9184575

[22] Lu M, Zhao B, Liu M, Wu L, Li Y, Zhai Y, et al. Pan-cancer analysis of SETD2 mutation and its association with the efficacy of immunotherapy. NPJ Precis Oncol. 2021;5:51. 34127768 10.1038/s41698-021-00193-0 PMC8203790

[23] Zhang YL, Sun JW, Xie YY, Zhou Y, Liu P, Song JC, et al. Setd2 deficiency impairs hematopoietic stem cell self-renewal and causes malignant transformation. Cell Res. 2018;28:476–90. 29531312 10.1038/s41422-018-0015-9 PMC5939047

[24] Zhou Y, Yan X, Feng X, Bu J, Dong Y, Lin P, et al. Setd2 regulates quiescence and differentiation of adult hematopoietic stem cells by restricting RNA polymerase II elongation. Haematologica. 2018;103:1110–23. 29650642 10.3324/haematol.2018.187708 PMC6029524

[25] Weng Y, Xue J, Niu N. SETD2 in cancer: functions, molecular mechanisms, and therapeutic regimens. Cancer Biol Med. 2024;21:725–30. 39302028 10.20892/j.issn.2095-3941.2024.0201 PMC11414219

[26] Ji Z, Sheng Y, Miao J, Li X, Zhao H, Wang J, et al. The histone methyltransferase Setd2 is indispensable for V(D)J recombination. Nat Commun. 2019;10:3353. 31350389 10.1038/s41467-019-11282-x PMC6659703

[27] Chu SH, Chabon JR, Matovina CN, Minehart JC, Chen BR, Zhang J, et al. Loss of H3K36 methyltransferase SETD2 impairs V(D)J recombination during lymphoid development. iScience. 2020;23:100941. 32169821 10.1016/j.isci.2020.100941 PMC7066224

[28] Ding Z, Cai T, Tang J, Sun H, Qi X, Zhang Y, et al. Setd2 supports GATA3(+)ST2(+) thymic-derived Treg cells and suppresses intestinal inflammation. Nat Commun. 2022;13:7468. 36463230 10.1038/s41467-022-35250-0 PMC9719510

[29] Chang J, Ji X, Deng T, Qiu J, Ding Z, Li Z, et al. Setd2 determines distinct properties of intestinal ILC3 subsets to regulate intestinal immunity. Cell Rep. 2022;38:110530. 35294891 10.1016/j.celrep.2022.110530

[30] Liu M, Rao H, Liu J, Li X, Feng W, Gui L, et al. The histone methyltransferase SETD2 modulates oxidative stress to attenuate experimental colitis. Redox Biol. 2021;43:102004. 34020310 10.1016/j.redox.2021.102004 PMC8141928

[31] Chen K, Liu J, Liu S, Xia M, Zhang X, Han D, et al. Methyltransferase SETD2-mediated methylation of STAT1 is critical for interferon antiviral activity. Cell. 2017;170:492–506.e14. 28753426 10.1016/j.cell.2017.06.042

[32] Mackenzie KJ, Carroll P, Martin CA, Murina O, Fluteau A, Simpson DJ, et al. cGAS surveillance of micronuclei links genome instability to innate immunity. Nature. 2017;548:461–5. 28738408 10.1038/nature23449 PMC5870830

[33] Dunphy G, Flannery SM, Almine JF, Connolly DJ, Paulus C, Jonsson KL, et al. Non-canonical activation of the DNA sensing adaptor STING by ATM and IFI16 mediates NF-kappaB signaling after nuclear DNA damage. Mol Cell. 2018;71:745–60.e5. 30193098 10.1016/j.molcel.2018.07.034 PMC6127031

[34] van de Rijn M, Heimfeld S, Spangrude GJ, Weissman IL. Mouse hematopoietic stem-cell antigen Sca-1 is a member of the Ly-6 antigen family. Proc Natl Acad Sci USA. 1989;86:4634–8. 2660142 10.1073/pnas.86.12.4634 PMC287325

[35] Essers MA, Offner S, Blanco-Bose WE, Waibler Z, Kalinke U, Duchosal MA, et al. IFNalpha activates dormant haematopoietic stem cells in vivo. Nature. 2009;458:904–8. 19212321 10.1038/nature07815

[36] Baldridge MT, King KY, Boles NC, Weksberg DC, Goodell MA. Quiescent haematopoietic stem cells are activated by IFN-gamma in response to chronic infection. Nature. 2010;465:793–7. 20535209 10.1038/nature09135 PMC2935898

[37] Kang K, Park SH, Chen J, Qiao Y, Giannopoulou E, Berg K, et al. Interferon-gamma represses M2 gene expression in human macrophages by disassembling enhancers bound by the transcription factor MAF. Immunity. 2017;47:235–50.e4. 28813657 10.1016/j.immuni.2017.07.017 PMC5568089

[38] Xie Y, Sahin M, Sinha S, Wang Y, Nargund AM, Lyu Y, et al. SETD2 loss perturbs the kidney cancer epigenetic landscape to promote metastasis and engenders actionable dependencies on histone chaperone complexes. Nat Cancer. 2022;3:188–202. 35115713 10.1038/s43018-021-00316-3 PMC8885846

[39] Simon JM, Hacker KE, Singh D, Brannon AR, Parker JS, Weiser M, et al. Variation in chromatin accessibility in human kidney cancer links H3K36 methyltransferase loss with widespread RNA processing defects. Genome Res. 2014;24:241–50. 24158655 10.1101/gr.158253.113 PMC3912414

[40] Sawamiphak S, Kontarakis Z, Stainier DY. Interferon gamma signaling positively regulates hematopoietic stem cell emergence. Dev Cell. 2014;31:640–53. 25490269 10.1016/j.devcel.2014.11.007 PMC4371141

[41] Kim PG, Canver MC, Rhee C, Ross SJ, Harriss JV, Tu HC, et al. Interferon-alpha signaling promotes embryonic HSC maturation. Blood. 2016;128:204–16. 27095787 10.1182/blood-2016-01-689281 PMC4946201

[42] Stadtfeld M, Graf T. Assessing the role of hematopoietic plasticity for endothelial and hepatocyte development by non-invasive lineage tracing. Development. 2005;132:203–13. 15576407 10.1242/dev.01558

[43] Morcos MNF, Schoedel KB, Hoppe A, Behrendt R, Basak O, Clevers HC, et al. SCA-1 expression level identifies quiescent hematopoietic stem and progenitor cells. Stem Cell Rep. 2017;8:1472–8. 10.1016/j.stemcr.2017.04.012 PMC5469944 28506535

[44] Wolf FA, Hamey FK, Plass M, Solana J, Dahlin JS, Gottgens B, et al. PAGA: graph abstraction reconciles clustering with trajectory inference through a topology preserving map of single cells. Genome Biol. 2019;20:59. 30890159 10.1186/s13059-019-1663-x PMC6425583

[45] Hormaechea-Agulla D, Matatall KA, Le DT, Kain B, Long X, Kus P, et al. Chronic infection drives Dnmt3a-loss-of-function clonal hematopoiesis via IFNgamma signaling. Cell Stem Cell. 2021;28:1428–42.e6. 33743191 10.1016/j.stem.2021.03.002 PMC8349829

[46] Sallman DA, List A. The central role of inflammatory signaling in the pathogenesis of myelodysplastic syndromes. Blood. 2019;133:1039–48. 30670444 10.1182/blood-2018-10-844654 PMC7022316

[47] Zhao JL, Ma C, O’Connell RM, Mehta A, DiLoreto R, Heath JR, et al. Conversion of danger signals into cytokine signals by hematopoietic stem and progenitor cells for regulation of stress-induced hematopoiesis. Cell Stem Cell. 2014;14:445–59. 24561084 10.1016/j.stem.2014.01.007 PMC4119790

[48] Hernandez-Malmierca P, Vonficht D, Schnell A, Uckelmann HJ, Bollhagen A, Mahmoud MAA, et al. Antigen presentation safeguards the integrity of the hematopoietic stem cell pool. Cell Stem Cell. 2022;29:760–75.e10. 35523139 10.1016/j.stem.2022.04.007 PMC9202612

[49] Wei Y, Zheng H, Bao N, Jiang S, Bueso-Ramos CE, Khoury J, et al. KDM6B overexpression activates innate immune signaling and impairs hematopoiesis in mice. Blood Adv. 2018;2:2491–504. 30275007 10.1182/bloodadvances.2018024166 PMC6177657

[50] Abegunde SJ, Rauh MJ. Tet2-deficient bone marrow progenitors have a proliferative advantage in the presence of TNF-alpha and IFN-gamma: implications for clonal dominance in inflammaging and MDS. Blood. 2015;126:2850.

[51] Crow YJ, Casanova JL. Human life within a narrow range: the lethal ups and downs of type I interferons. Sci Immunol. 2024;9:eadm8185. 38968338 10.1126/sciimmunol.adm8185

[52] Yanez A, Coetzee SG, Olsson A, Muench DE, Berman BP, Hazelett DJ, et al. Granulocyte-monocyte progenitors and monocyte-dendritic cell progenitors independently produce functionally distinct monocytes. Immunity. 2017;47:890–902.e4. 29166589 10.1016/j.immuni.2017.10.021 PMC5726802

[53] Xu C, He J, Wang H, Zhang Y, Wu J, Zhao L, et al. Single-cell transcriptomic analysis identifies an immune-prone population in erythroid precursors during human ontogenesis. Nat Immunol. 2022;23:1109–20. 35761081 10.1038/s41590-022-01245-8

[54] Meisel M, Hinterleitner R, Pacis A, Chen L, Earley ZM, Mayassi T, et al. Microbial signals drive pre-leukaemic myeloproliferation in a Tet2-deficient host. Nature. 2018;557:580–4. 29769727 10.1038/s41586-018-0125-z PMC6238954

