# Supplementary material for: Setd2 ensures the establishment of a precise basal inflammatory state within murine hematopoietic stem/progenitor cells
Source: Cell Death Dis. 2025 Nov 6;16(1):799. doi: 10.1038/s41419-025-08110-0 (PMC12592349; doi:10.1038/s41419-025-08110-0)
Supplement: Supplementary file 1 — Supplementary Materials [file 41419_2025_8110_MOESM1_ESM.docx]

**Supplementary Figure**

**
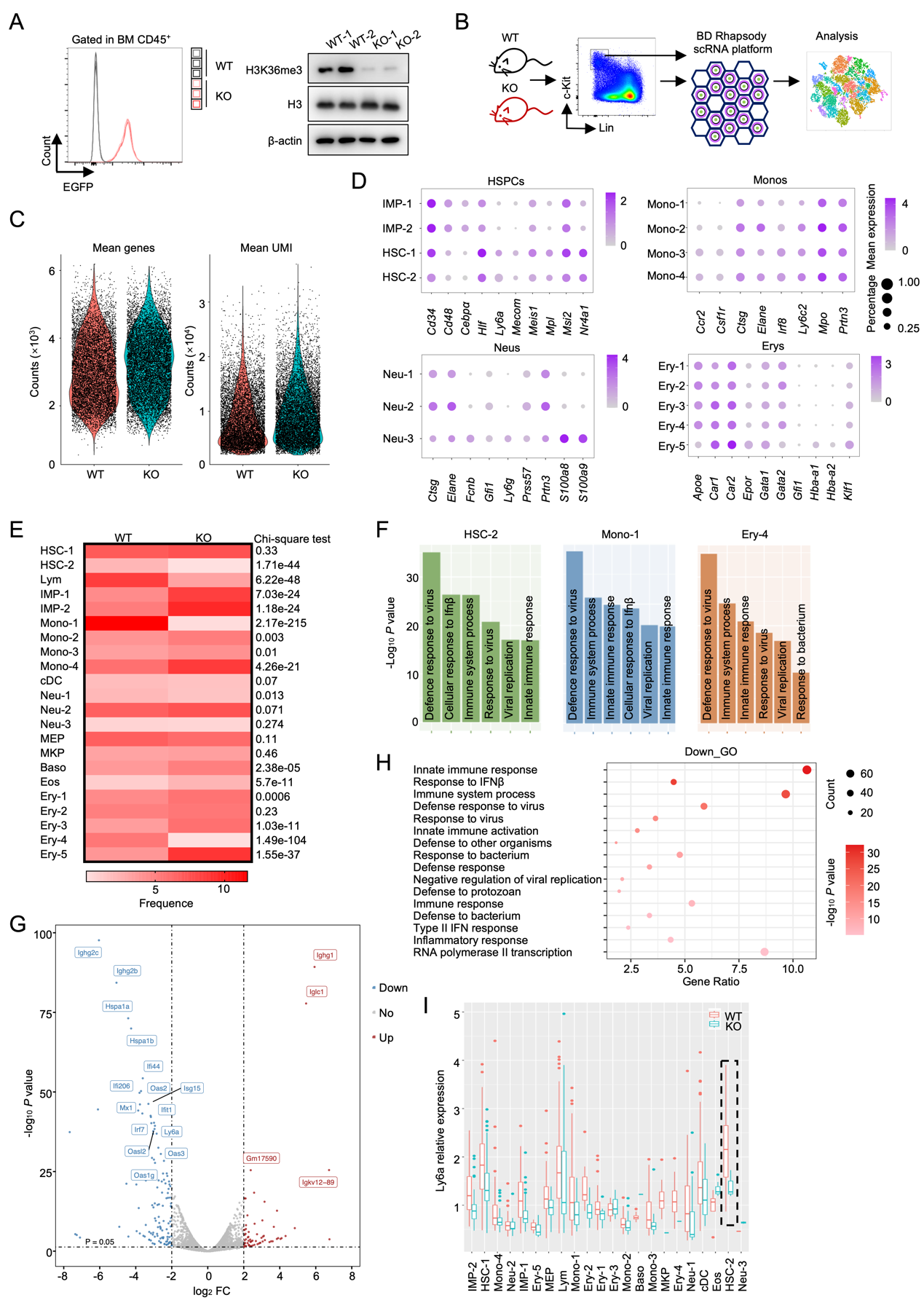
**

**Fig. S1. Molecular characterization of *Setd2* conditional knockout and scRNA-seq profiling of BM LKs, related to Fig. 1. (A)** Validation of *Setd2* conditional knockout: Flow cytometry of EGFP in BM CD45^+^ cells (left); Western blot of H3K36me3, H3, and β-actin expression in BM cells (right). **(B)** Schematic of scRNA-seq analysis for BM LKs. **(C)** Average gene counts (left) and UMI counts (right) per cell. **(D)** Bubble plot of marker genes used for cluster annotation. (**E**) Cluster frequency comparison between WT and KO samples. *P* values calculated by Chi-square test. **(F)** Top GO-BP terms for marker genes of HSC-2, Mono-1, Ery-4. **(G)** Volcano plot of DEGs in KO vs. WT (*P* < 0.05 and | log_2_ (FC) | ≥ 0.585). **(H)** Bubble plots of top enriched GO-BP terms for downregulated genes in KO. **(I)** Relative *Ly6a* expression per cluster.

**
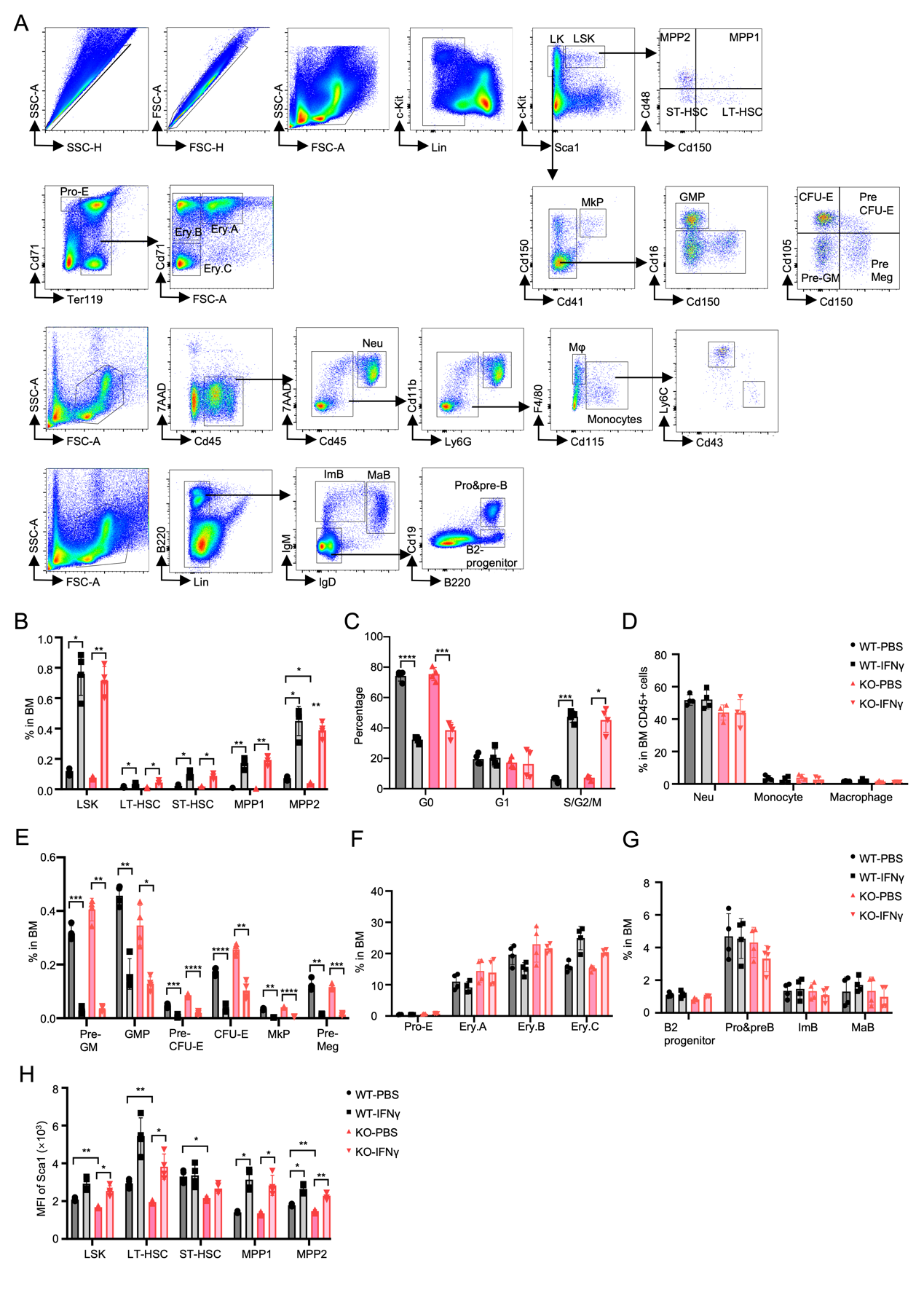
**

**Fig. S2. Acute hematopoietic compartment remodeling in response to high-dose IFNγ challenge, related to Fig. 2. (A)** Representative BM gating strategy. **(B-H)** Flow cytometry of BM from WT and KO mice 24h post PBS/IFNγ (10 μg) treatment. n = 4. **(B)** LSK and subset frequency in total BM. **(C)** Cell cycle analysis of LSKs. **(D)** Granulocyte-monocyte subset frequency in total BM **(E)** Hematopoietic progenitor subset frequency in total BM. **(F)** Erythroid subset frequency in total BM. **(G)** B-cell lineage subset frequency in total BM. **(H)** MFI quantification of Sca1 in LSKs and subsets.


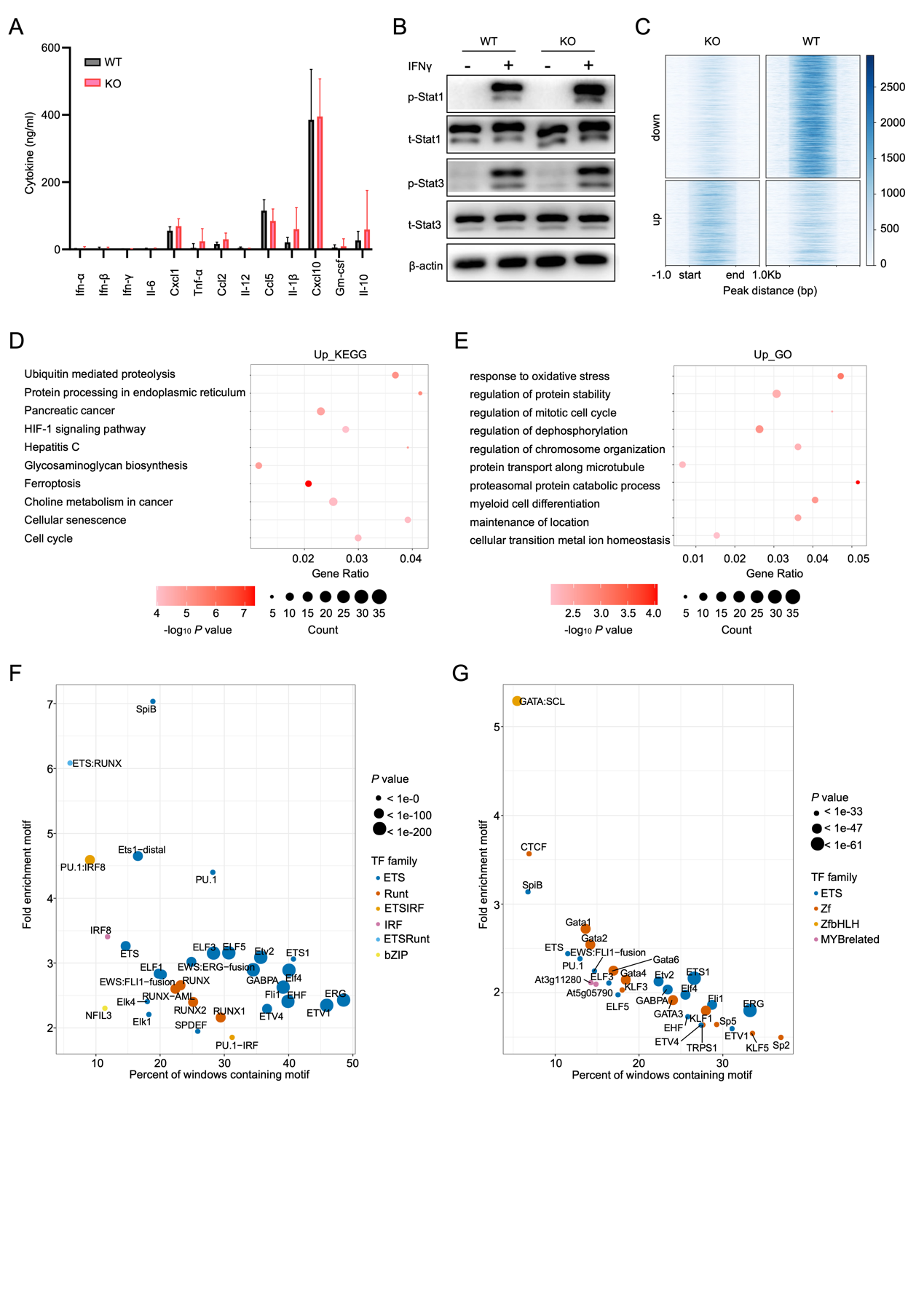


**Fig. S3.** **Systemic cytokine detection and epigenomic remodeling in *Setd2*-deficient LSKs, related to Fig. 3. (A)** Serum cytokine quantification in WT and KO mice. n = 6. **(B)** Western blot of sorted LKs treated with 100 ng/mL IFNγ. **(C)** Heatmaps of altered ATAC-seq peaks (± 1.0-Kb window) in KO vs. WT LSKs. **(D-E)** Bubble plots of enriched **(D)** KEGG pathways and **(E)** GO-BP terms for genes linked to increased ATAC-seq peaks in KO LSKs. **(F-G)** Top 30 enriched TF motifs in regions with **(F)** decreased and **(G)** increased ATAC-seq accessibility in KO vs. WT LSKs.

**
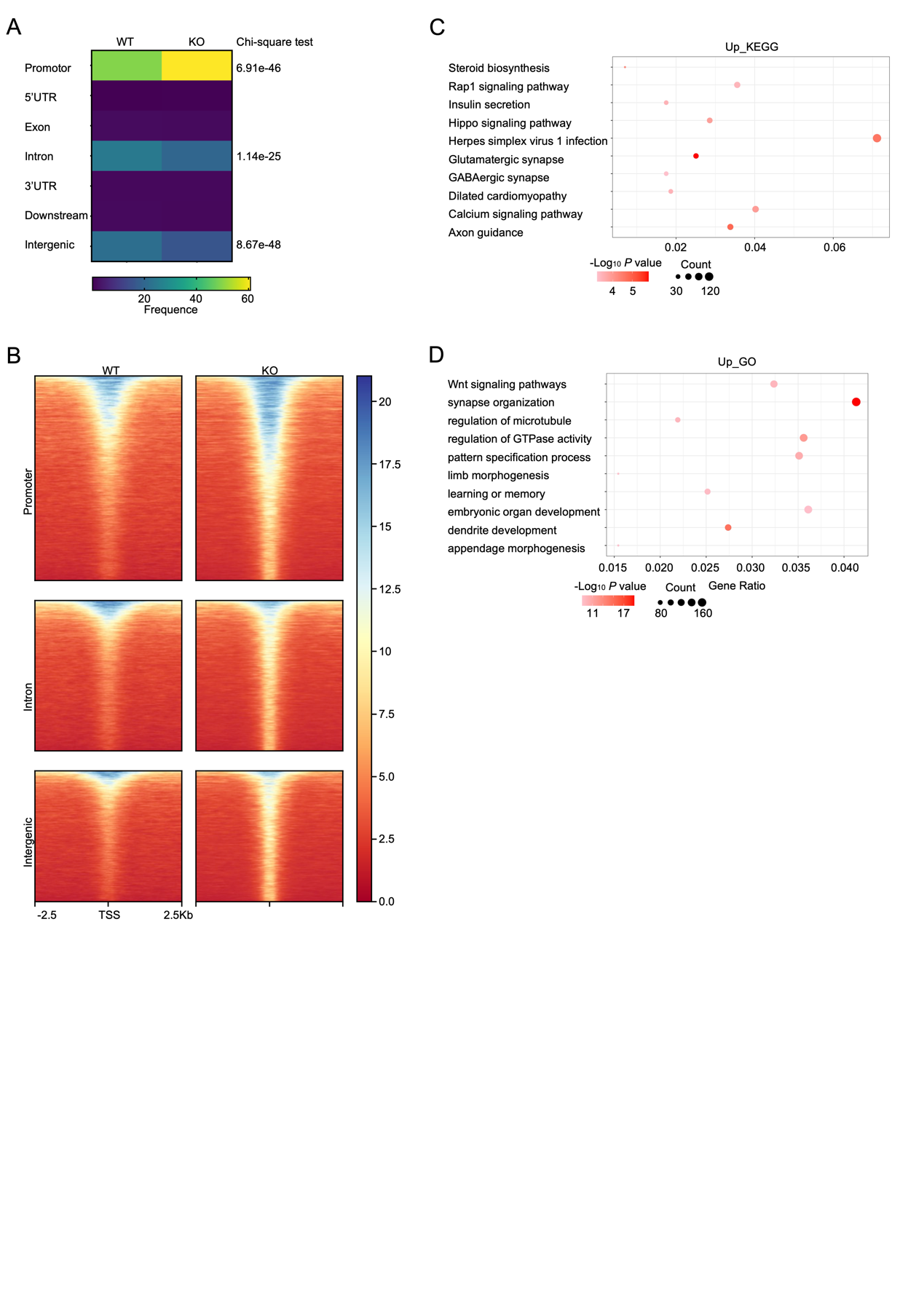
**

**Fig. S4. Genomic annotation of *Setd2*-dependent chromatin accessibility and enhancer landscapes, related to Fig. 4. (A)** Genomic distribution frequency of differential ATAC-seq peaks, *P* values calculated by Chi-square test. **(B)** Heatmaps of H3K27ac peaks (± 2.5-Kb window) grouped by genomic localization. **(C-D)** Bubble plots of enriched **(C)** KEGG pathways and **(D)** GO-BP terms for genes linked to increased H3K27ac peaks.

**
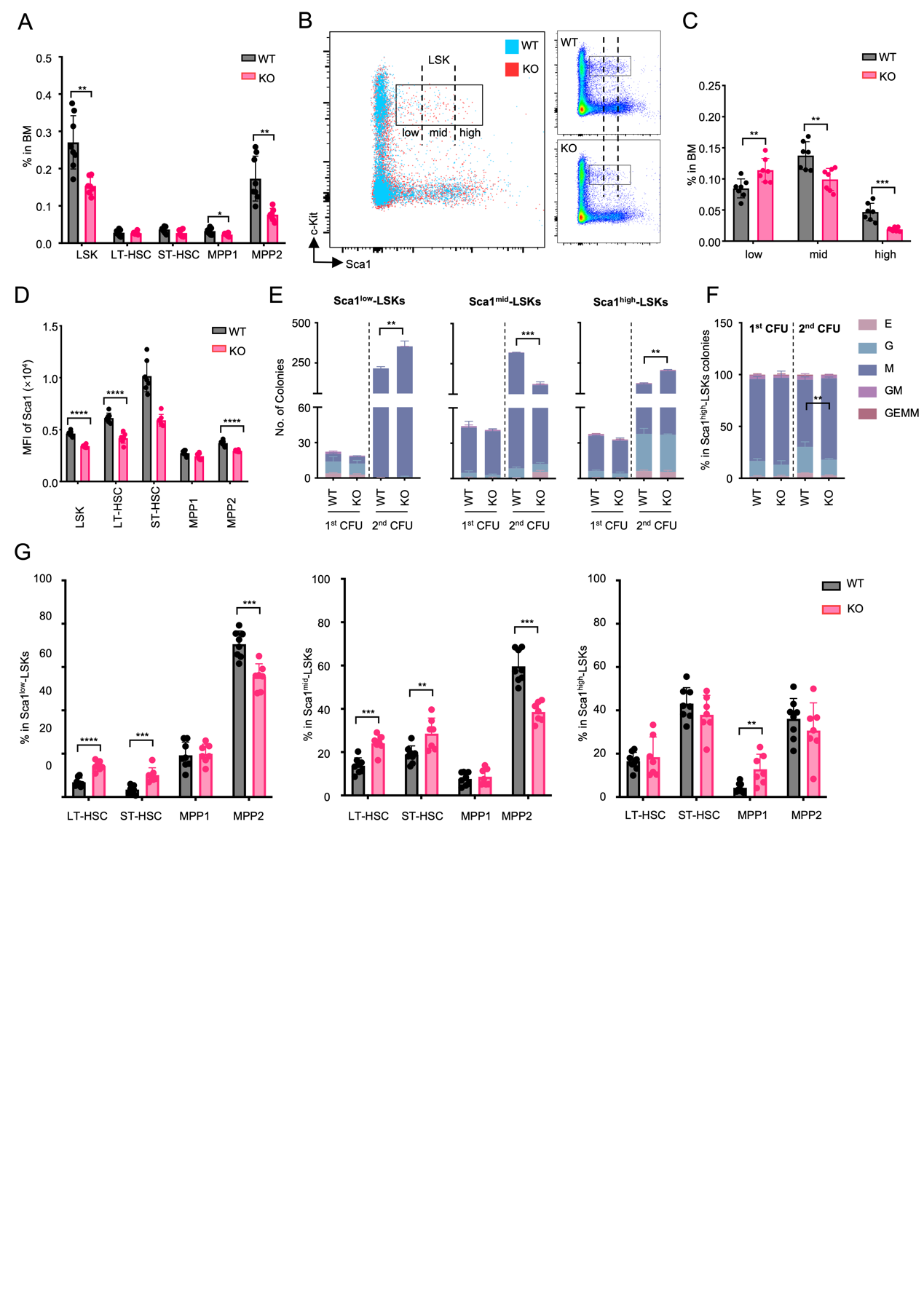
**

**Fig. S5. Functional heterogeneity of three Sca1-marked LSK subpopulations, related to Fig. 5. (A)** LSK and subset frequency in total BM. **(B-D)** Flow cytometry of BM from WT and vav-Cre *Setd2*-KO mice. n = 7. **(B)** Representative Sca1^low^, Sca1^mid^, and Sca1^high^-LSK gating. **(C)** Frequency of Sca1^low^, Sca1^mid^, and Sca1^high^-LSKs in total BM. **(D)** MFI quantification of Sca1 in LSKs and subsets. **(E)** Serial CFU assay of Sca1^low^, Sca1^mid^ and Sca1^high^-LSKs from WT and KO mice. n = 3. **(F)** Colony type percentage in WT and KO Sca1^high^-LSKs. **(G)** Frequency of LSK subsets within Sca1^low^, Sca1^mid^ and Sca1^high^ populations. WT, n = 8; KO, n = 7.

**
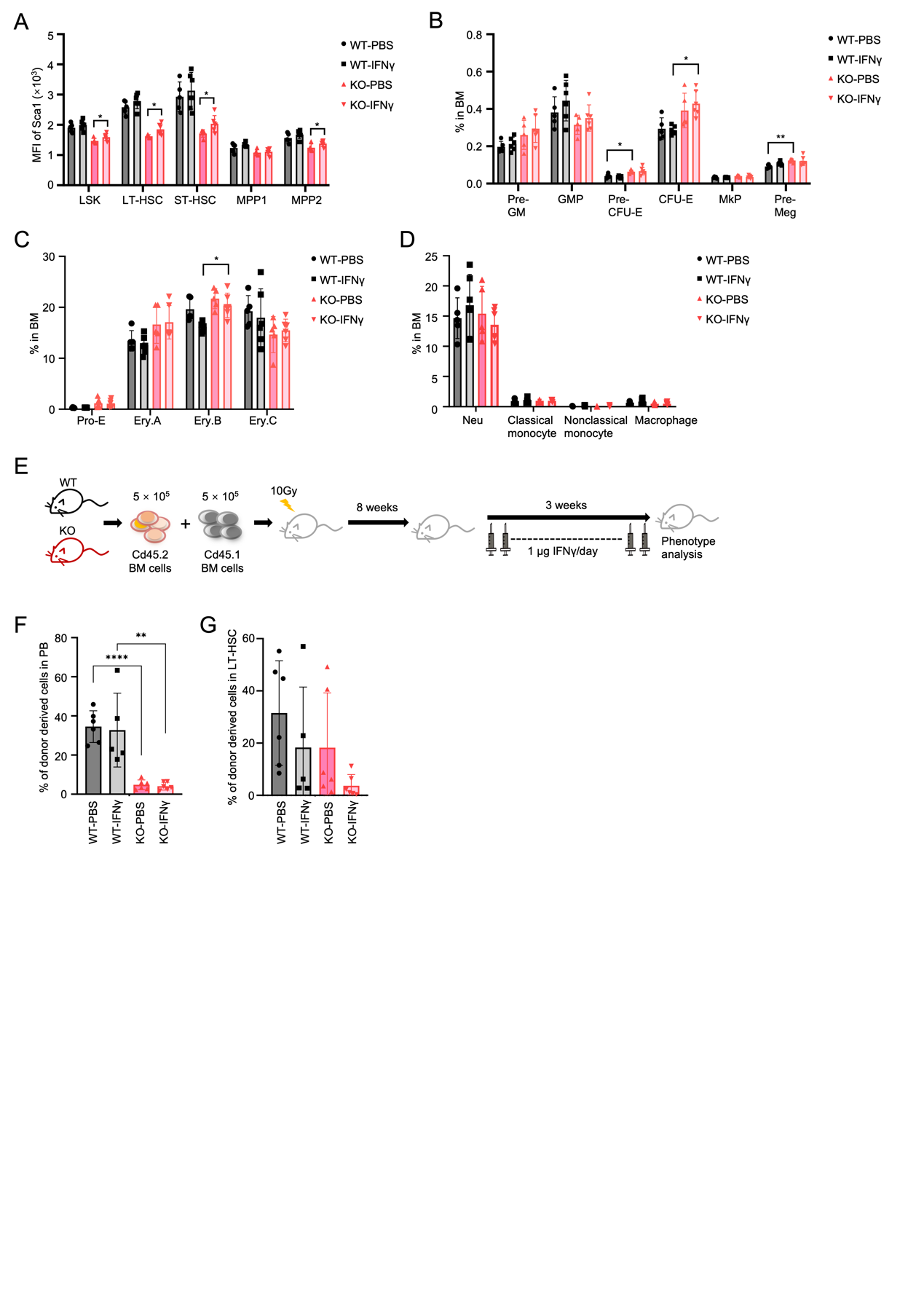
**

**Fig. S6. Chronic low-dose IFNγ exposure remodels hematopoietic compartments in primary and transplanted mice, related to Fig. 6. (A-D)** Flow cytometry of BM from primary WT (PBS, n=5; IFNγ, n=6) and KO (PBS, n=5; IFNγ, n=6) mice after 3-week IFNγ treatment (1 μg/day). **(A)** MFI quantification of Sca1 in LSKs and subsets. **(B)** Hematopoietic progenitor subset frequency in total BM. **(C)** Erythroid subset frequency in total BM. **(D)** Granulocyte-monocyte subset frequency in total BM. **(E)** Schematic representation of cBMT. **(F-G)** Flow cytometry of BM from transplanted WT (PBS, n=6; IFNγ, n=5) and KO (PBS, n=6; IFNγ, n=6) mice after 3-week IFNγ treatment (1 μg/day). **(F)** Donor-derived cell frequency in PB. **(G)** Donor-derived cell frequency in BM LT-HSC.
