# Supplementary figures and images for: Setd2 ensures the establishment of a precise basal inflammatory state within murine hematopoietic stem/progenitor cells
Source: Cell Death Dis. 2025 Nov 6;16(1):799. doi: 10.1038/s41419-025-08110-0 (PMC12592349; doi:10.1038/s41419-025-08110-0)

p-STAT1

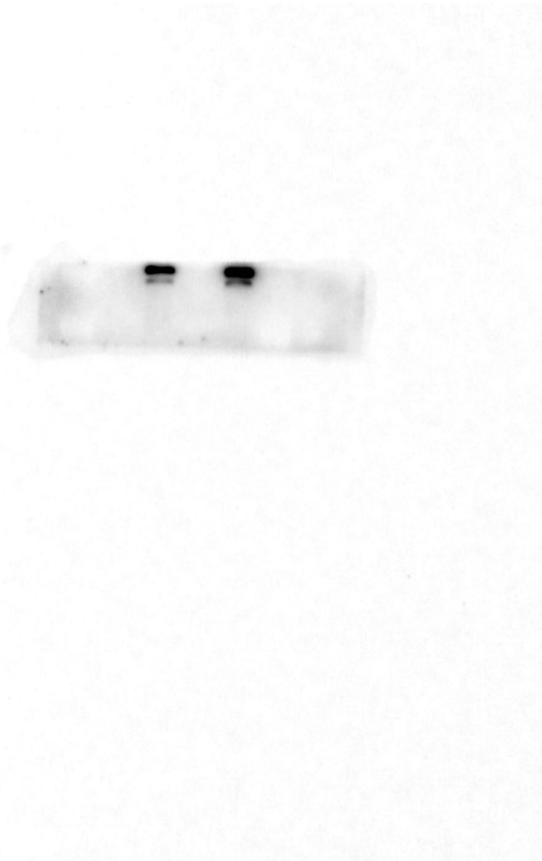

t-STAT1

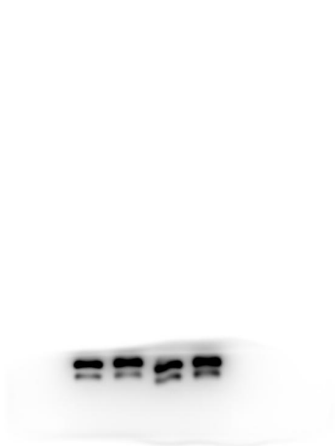

p-STAT3

t-STAT3

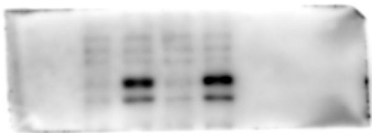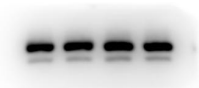

$\beta$ -actin

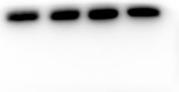

H3

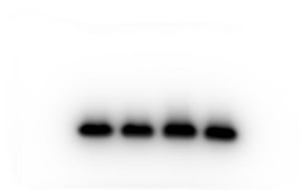

H3K36me3

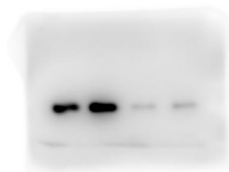

$\beta$ -actin

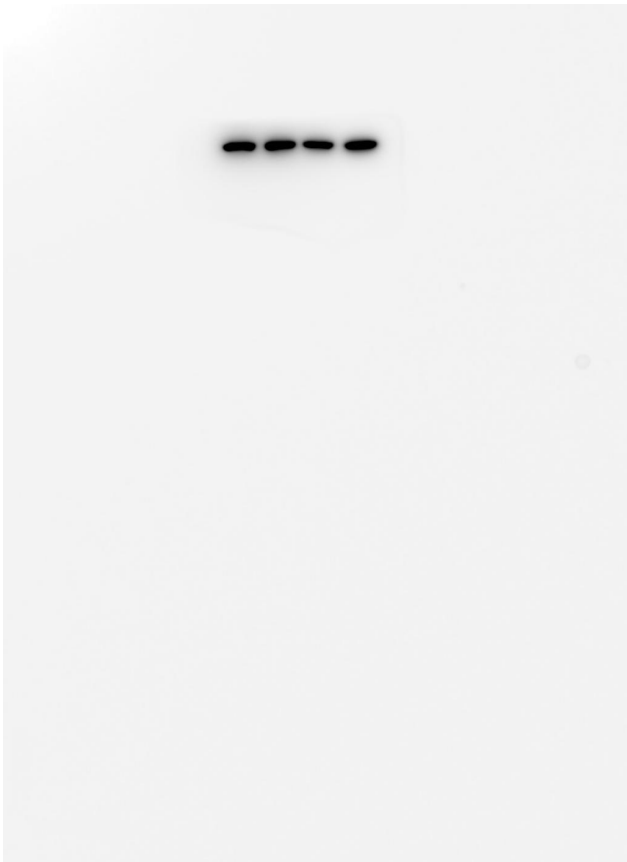

Supplement: Supplementary file 5 — Supplementary WB results [file 41419_2025_8110_MOESM5_ESM.pdf]
